# Supplementary material for: A high-affinity split-HaloTag for live-cell protein labeling
Source: Nat Commun. 2026 Mar 25;17:2865. doi: 10.1038/s41467-026-71032-8 (PMC13022382; doi:10.1038/s41467-026-71032-8)
Supplement: Supplementary file 1 — Supplementary Information [file 41467_2026_71032_MOESM1_ESM.pdf]

Supplementary Information for

A high-affinity split-HaloTag for live-cell protein labeling

Yin-Hsi Lin[1,2], Julian Kompa[1], De-en Sun[1], Runyu Mao[1], Birgit Koch[1], Konstantin Hinnah[1], Jonas Wilhelm[1], Natascha Franz[1], Stefanie Kühn[1], Tanja Menche[1], Abdinasir Adow[1], Paula Breuer[1], Julien Hiblot[1*], Kai Johnsson[1,3*]

[1] Department of Chemical Biology, Max Planck Institute for Medical Research, Jahnstrasse 29, 69120 Heidelberg, Germany.
[2] Institute of Bioengineering (BioE), École Polytechnique Fédérale de Lausanne (EPFL), 1015 Lausanne, Switzerland
[3] Institute of Chemical Sciences and Engineering (ISIC), École Polytechnique Fédérale de Lausanne (EPFL), 1015 Lausanne, Switzerland

*email: julien.hiblot@mr.mpg.de; johnsson@mr.mpg.de

Table of content

Table of content **2**

Supplementary Figures **3**

Supplementary Fig 1. cpHaloΔ engineering via high-throughput screening. 3
Supplementary Fig 2. Selection of cpHaloΔ yeast libraries by FACS. 4
Supplementary Fig 3. Characterization of the EC_{50} and labeling kinetics of engineered split-HaloTag pairs. 5
Supplementary Fig 4. cpHaloΔ background labeling activity in cells. 6
Supplementary Fig 5. Melting temperature of cpHaloΔ2 and cpHaloΔ3. 6
Supplementary Fig 6. Comparison of fluorescence intensity of the labeled split-HaloTag pairs and HaloTag. 7
Supplementary Fig 7. Biophysical characterization of split-HaloTag pairs. 8
Supplementary Fig 8. Split-HaloTag structures predicted by AlphaFold3. 10
Supplementary Fig 9. Intensity profiles across cells expressing Hpep-tagged POIs. 12
Supplementary Fig 10. Performance of split-HaloTag in mammalian cells. 14
Supplementary Fig 11. Assessment of Hpep11 performance at different insertion sites in EGFP. 15
Supplementary Fig 12. The performance of Hpep variants for endogenous protein labeling. 16
Supplementary Fig 13. Comparison of split-HaloTag and HaloTag performance in live-cell imaging of endogenously tagged TOM20. 18
Supplementary Fig 14. Fluorescent SLP substrates used in this study. 19
Supplementary Fig 15. HPLC-HRMS analysis of the biotinylated Hpep9. 20
Supplementary Fig 16. HPLC-HRMS analysis of the biotinylated Hpep11. 21
Supplementary Fig 17. HPLC-HRMS analysis of the TMR-conjugated Hpep9. 22
Supplementary Fig 18. HPLC-HRMS analysis of the TMR-conjugated Hpep11. 23
Supplementary Fig 19. SDS-PAGE analysis of the recombinant cpHaloΔ proteins. 24
Supplementary Fig 20. Gating strategy for yeast library screening. 24
Supplementary Fig 21. Gating strategy to enrich Hpep11-integrated cells. 25

Supplementary Tables **26**

Supplementary Table 1. EC_{50} values of Hpep variants for the parental cpHaloΔ. 26
Supplementary Table 2. Affinity measurement of labeled-cpHaloΔ3 to biotin-Hpep conjugates. 26
Supplementary Table 3. CRISPR/Cas9 KI target information. 27
Supplementary Table 4. Fluorescence lifetime of split-HaloTag pairs. 28
Supplementary Table 5. Medium and buffers used in this study. 28
Supplementary Table 6. Spectral properties, reactive protein tags and full names of the fluorescent ligands used in this study. 29
Supplementary Table 7. Spectral properties of fluorophores used in this work. 29
Supplementary Table 8. PCR reaction recipe for cpHaloΔ library generation. 29
Supplementary Table 9. PCR reaction protocol for cpHaloΔ library generation. 30
Supplementary Table 10. Digestion reaction recipe for cpHaloΔ library generation. 30
Supplementary Table 11. Ligation reaction recipe for cpHaloΔ library generation. 30
Supplementary Table 12. FACS and flow cytometry analysis. 31
Supplementary Table 13. Sorting strategy for N-terminal extension cpHaloΔ library. 31
Supplementary Table 14. Sorting strategy for C-terminal extension cpHaloΔ library. 31
Supplementary Table 15. Primers for library generation and NGS sample preparation. 31
Supplementary Table 16. Stable cell lines generated in this study. 32
Supplementary Table 17. U2OS transient transfection and associated experimental figures. 33
Supplementary Table 18. Confocal image acquisition parameters. 34
Supplementary Table 19. Confocal microscope laser powers. 39
Supplementary Table 20. STED image acquisition parameters. 40
Supplementary Table 21. STED microscope laser powers. 41
Supplementary Table 22. Summary of binding and fluorescence properties of Hpep variants in complex with cpHaloΔ3. 41

Protein sequences **42**

Expression in *E.coli* **42**

Expression on yeast surface **42**

Expression in mammalian cells **42**

References **45**

Supplementary Figures

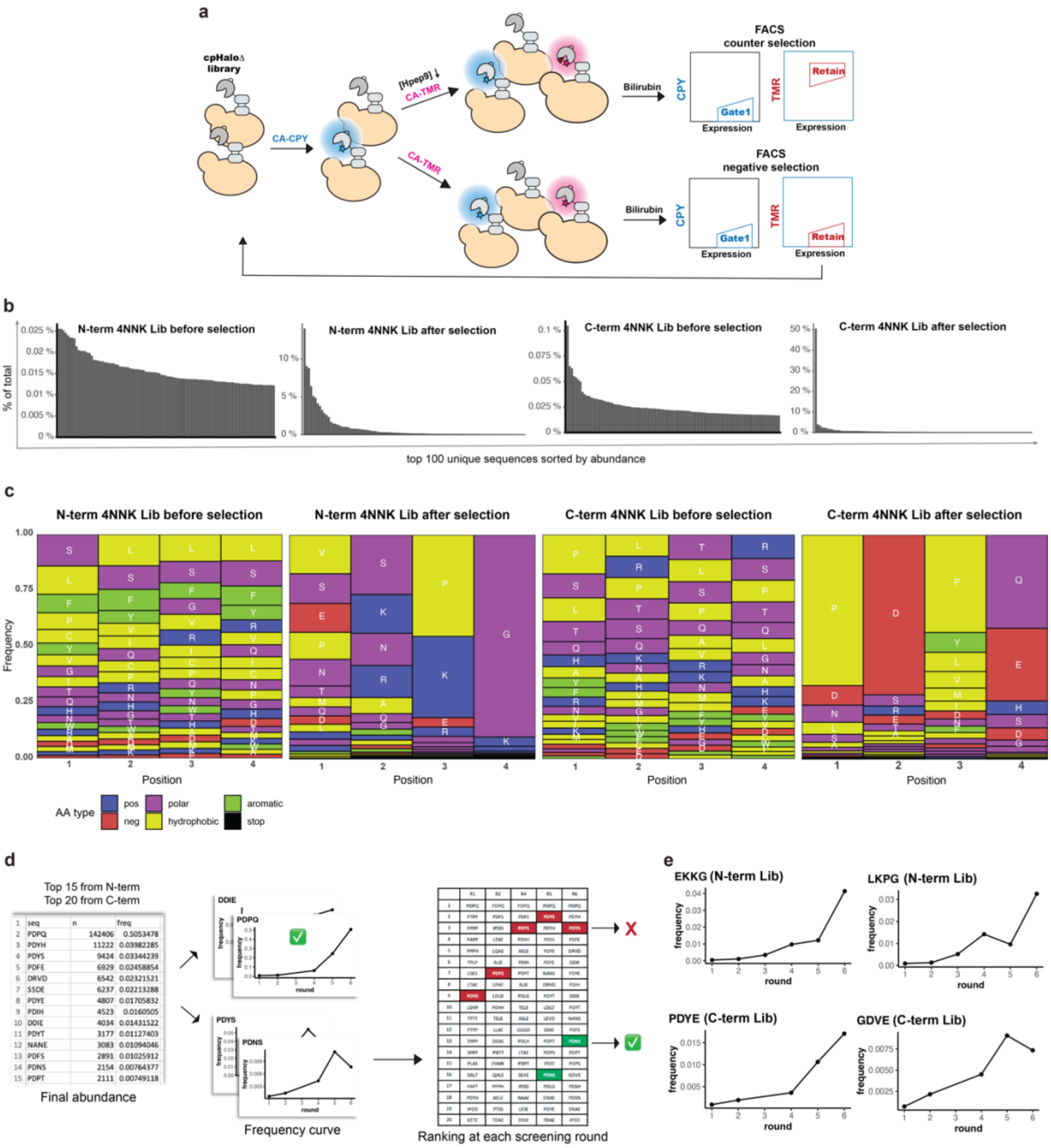

Supplementary Fig 1. cpHaloΔ engineering via high-throughput screening.

(a), Screening procedure for yeast surface display cpHaloΔ libraries. A negative sort was conducted exclusively (at 5th round of N-term NNK library screening) when no discernible distinction was noted between the TMR labeling signal in the presence and absence of Hpep9. (b), The distribution of the top 100 sequences in each library, generated from the NGS sequencing results. It revealed substantial sequence enrichment throughout the screening process. This subset accounted for only 1.6 % and 2.6 % of the entire N-term NNK and C-term NNK library, respectively, before screening, but represented 95 % and 96 % of each library after screening. (c), Amino acid frequency plots illustrating the enrichment of specific residues at each position after multiple rounds of FACS sorting. (d), An overview of the strategy used for identifying promising cpHaloΔ variants for further in vitro characterization. Frequency curves and the abundance ranking were used as two key parameters to evaluate the fitness of the each cpHaloΔ candidate. (e), Frequency curves of the top two performing cpHaloΔ variants from each library in the subsequent *in vitro* assay. (Supplementary Fig 3).

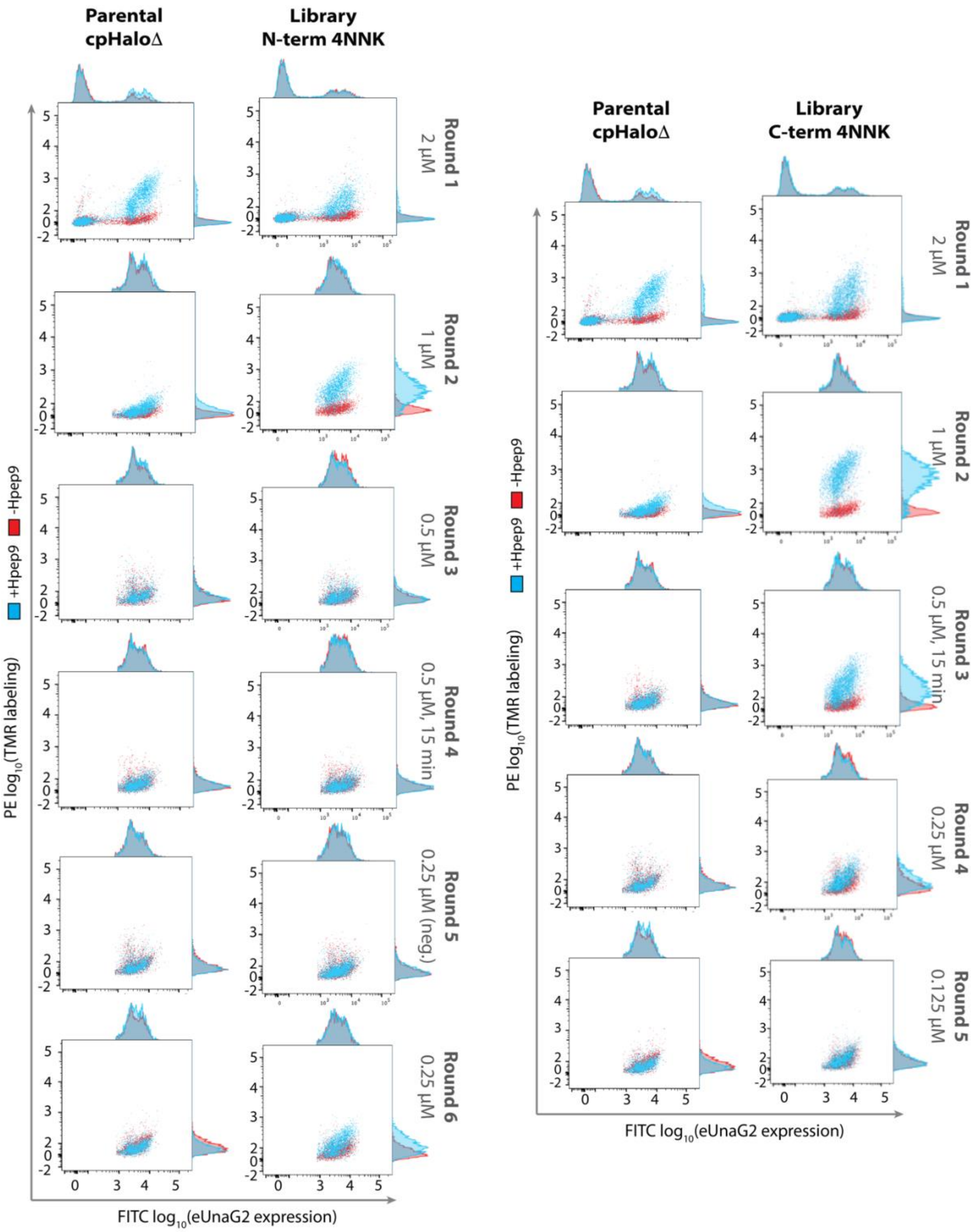

Supplementary Fig 2. Selection of cpHaloΔ yeast libraries by FACS.

FACS plots of yeast libraries at each round of selection for cpHaloΔ yeast library with N and C-terminal 4NNK extension. The screening was conducted for 5-6 rounds until no TMR labeling could be detected at the low Hpep9 concentrations.

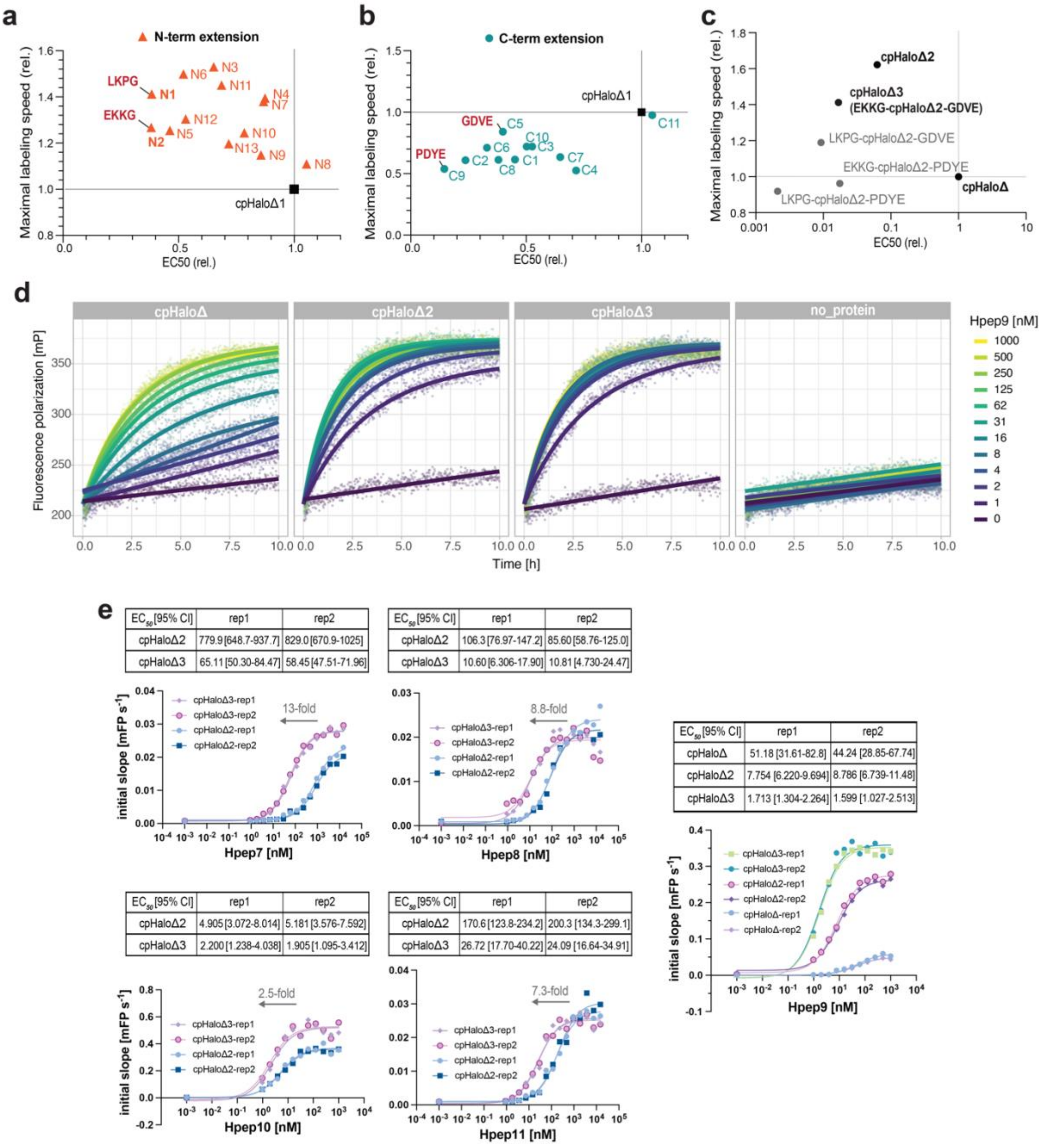

EC_{50} [95% CI]	rep1	rep2
cpHaloΔ2	779.9 [648.7-937.7]	829.0 [670.9-1025]
cpHaloΔ3	65.11 [50.30-84.47]	58.45 [47.51-71.96]

EC_{50} [95% CI]	rep1	rep2
cpHaloΔ2	106.3 [76.97-147.2]	85.60 [58.76-125.0]
cpHaloΔ3	10.60 [6.306-17.90]	10.81 [4.730-24.47]

EC_{50} [95% CI]	rep1	rep2
cpHaloΔ	51.18 [31.61-82.8]	44.24 [28.85-67.74]
cpHaloΔ2	7.754 [6.220-9.694]	8.786 [6.739-11.48]
cpHaloΔ3	1.713 [1.304-2.264]	1.599 [1.027-2.513]

EC_{50} [95% CI]	rep1	rep2
cpHaloΔ2	4.905 [3.072-8.014]	5.181 [3.576-7.592]
cpHaloΔ3	2.200 [1.238-4.038]	1.905 [1.095-3.412]

EC_{50} [95% CI]	rep1	rep2
cpHaloΔ2	170.6 [123.8-234.2]	200.3 [134.3-299.1]
cpHaloΔ3	26.72 [17.70-40.22]	24.09 [16.64-34.91]

Supplementary Fig 3. Characterization of the EC_{50} and labeling kinetics of engineered split-HaloTag pairs.

(**a-c**), Performance of the selected cpHaloΔ hits as recombinant proteins, validated by their labeling affinity to Hpep9 and the relative labeling speed in presence of Hpep9 compared to parental cpHaloΔ. Most of the selected variants showed a decrease in EC_{50}, used as a proxy for binding affinity, and most of the N-terminal extended variants showed a slight improvement in TMR labeling kinetics. All the measurements were performed on the same day with freshly purified proteins with technical duplicates. The labeling kinetics were measured with 20 nM cpHaloΔ protein, 4 nM CA-TMR with a titration of Hpep9 from 1 µM to 0.98 nM for determining EC_{50} and maximal labeling speed. (**c**), The performance of combinatorial cpHaloΔ variants created by combining the best-performing N- and C-terminal extension sequences with the stabilization mutations from cpHaloΔ2 as reported previously[1]. (**d**), TMR [0.5 nM] labeling traces at 37 °C of the three cpHaloΔ variants [2.5 nM] with Hpep9 at varying concentration [from 1 µM to 0.98 nM]. (**e**), cpHaloΔ3 showed higher affinity (lower EC_{50}) to Hpep7-11 compared to cpHaloΔ2. In each graph, the value above the arrow represents the fold-change in EC_{50} from cpHaloΔ2 to cpHaloΔ3.

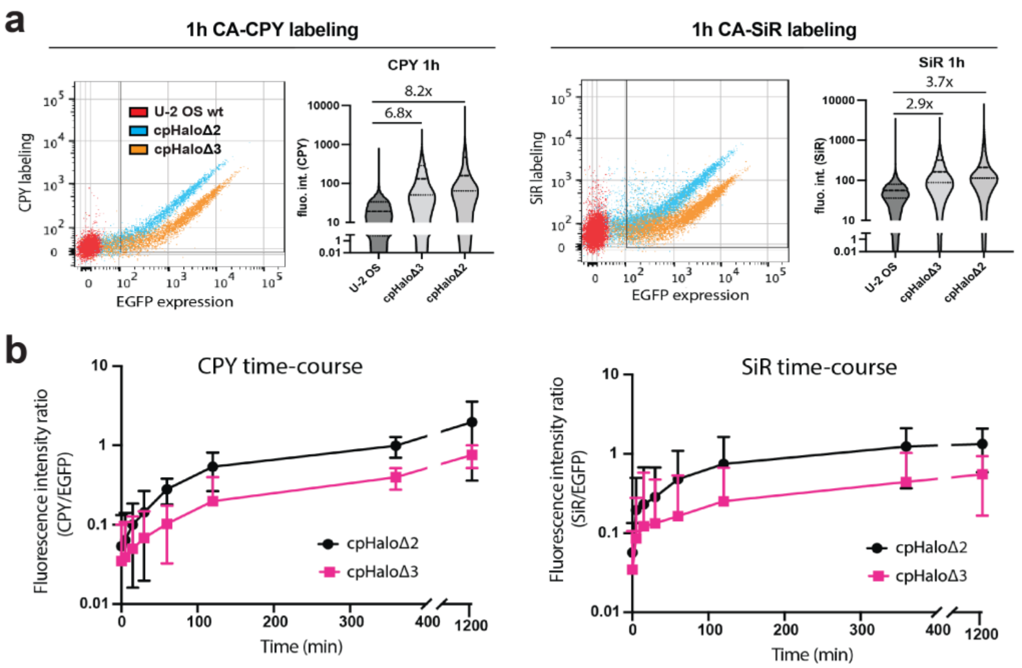

Supplementary Fig 4. cpHaloΔ background labeling activity in cells.

Investigation of the background labeling activity of cpHaloΔ variants (EGFP-GSG-cpHaloΔ) in U2OS cells via flow cytometry analysis. **(a),** Flow cytometry plots and the violin plot representations of the signal from cpHaloΔ expressing cells after one hour of labeling with 100 nM CA-CPY (upper) or CA-SiR (lower). Although still higher than U2OS blank cells, cpHaloΔ3 showed a lower background labeling activity for both dyes in comparison to cpHaloΔ2. **(b),** Labeling traces of U2OS stable cell lines expressing cpHaloΔ variants. Labeling was conducted with 100 nM CA-CPY or 100 nM CA-SiR for a period of 21 hours. Mean and s.d. of the EGFP normalized HaloTag ligand labeling ratio are shown here at each time point, calculated from n > 2500 cells.

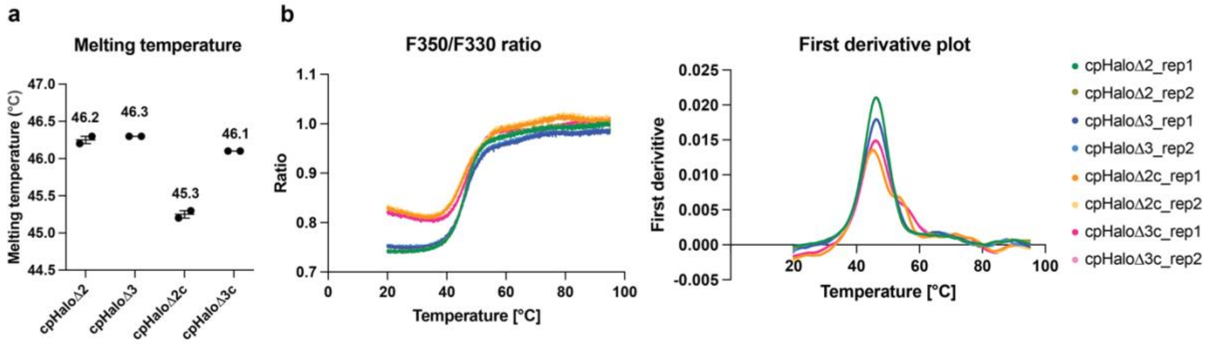

Supplementary Fig 5. Melting temperature of cpHaloΔ2 and cpHaloΔ3.

Melting temperature of cpHaloΔ2 and cpHaloΔ3 were determined by nanoDSF. cpHaloΔX stands for His-tagged proteins and cpHaloΔXc represents proteins without His-tag, which were prepared via TEV-cleavage, followed by reverse-IMAC and SEC purification. **(a),** Average melting temperature for each variant from technical duplicate measurements are indicated above each variant. **(b),** Intrinsic fluorescence intensity ratios at 350 nm and 330 nm were plotted as a function of temperature, ranging from 20°C to 95°C (left). First derivative of the ratio was plotted against temperature (right).

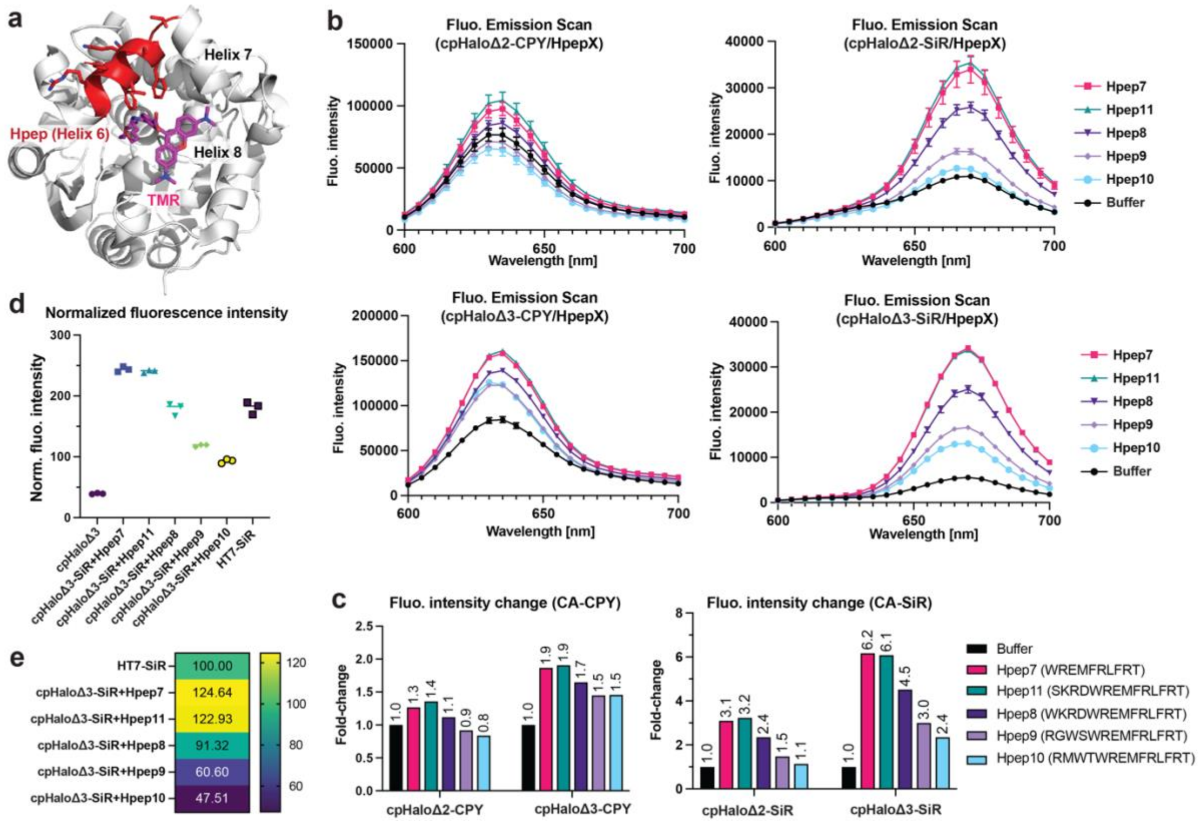

Supplementary Fig 6. Comparison of fluorescence intensity of the labeled split-HaloTag pairs and HaloTag.

(**a**), Crystal structure of HaloTag-TMR (PDB ID: 6Y7A), highlighting Hpep and its participation in forming the TMR binding site (helices 6-8). HaloTag is represented in grey, Hpep in red and TMR in magenta. (**b**), Fluorescence intensity comparison of labeled cpHaloΔ2, cpHaloΔ3 in the absence and presence of five Hpep variants. CPY-labeled cpHaloΔ (upper) and SiR-labeled cpHaloΔ (lower) in general showed a fluorescence increase upon binding to Hpep, except for the combination of CPY-labeled cpHaloΔ2 with Hpep9 and Hpep10 bound. All measurement were done on the same day with technical triplicates. Error bars representing 95 % confidence intervals for technical triplicates. (**c**), Quantifying the fluorescence intensity fold-change for each cpHaloΔ/Hpep pair in comparison to cpHaloΔ alone. (**d**), Comparison of the fluorescence intensity of cpHaloΔ3 complex to that of the intact HaloTag by calculating normalized fluorescence intensity, where the intensity values were normalized by the concentration of the labeled-cpHaloΔ protein present in the assay. (**e**), Relative fluorescence intensity comparison of the labeled-cpHaloΔ3 complex with that of the intact HaloTag.

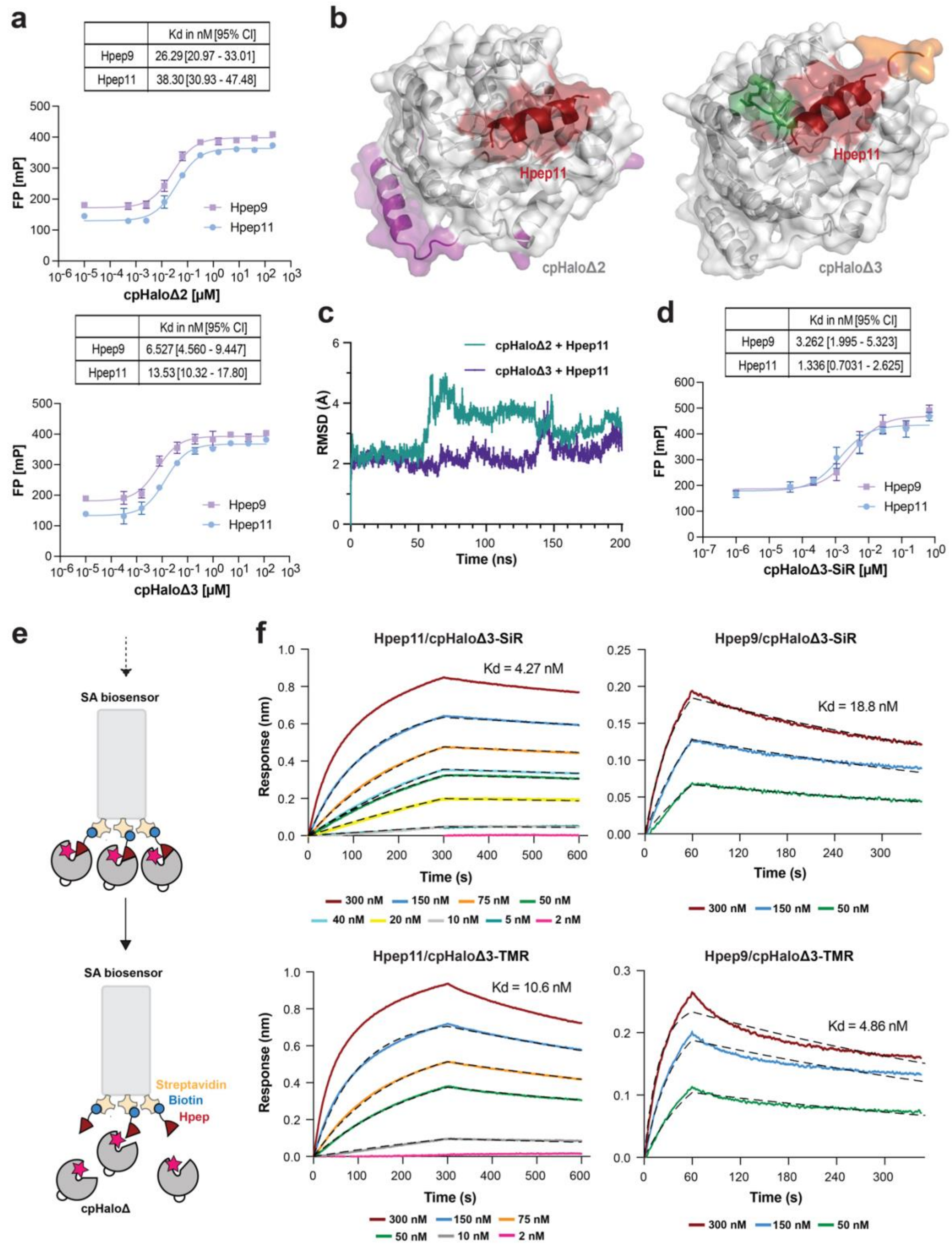

Supplementary Fig 7. Biophysical characterization of split-HaloTag pairs.

(**a**), K_d determination by FP assay. The binding affinity of cpHaloΔ3 to Hpep9 and Hpep11 was 4- and 2.8-fold higher, respectively, than that of cpHaloΔ2. cpHaloΔ was titrated against chemically synthesized TMR-Hpep conjugates [1 nM], and fluorescence polarization was measured at 25 °C. Mean and s.d. calculated from technical triplicates were plotted. (**b**), AlphaFold3[2] model of cpHaloΔ2 and cpHaloΔ3 in complex with Hpep11. cpHaloΔ2 was engineered from cpHaloΔ1 through introduction of stabilization mutations (purple) including four mutations and the redesigned CP-linker. cpHaloΔ3 was created by introducing EKKG (orange) and GDVE (forest) to N- and C-terminus to cpHaloΔ2, respectively. Hpep11 is highlighted in red. (**c**), MD simulation of Hpep11 in complex with cpHaloΔ2 or cpHaloΔ3. Hpep11 showed a higher root-mean-square deviation (RMSD) and greater conformational flexibility when bound to cpHaloΔ2, with pronounced fluctuations occurring after approximately 50 ns. In contrast, Hpep11/cpHaloΔ3 complex exhibited significantly reduced conformational fluctuations, maintaining structural stability for at least 140 ns. (**d**), K_d determination of SiR-labeled cpHaloΔ3 with Hpep9 and Hpep11 by FP assay, as described in (a). (**e**), A schematic of binding kinetics analysis of labeled cpHaloΔ3 and Hpep using BLI assay, highlighting the association and dissociation step. Chemically synthesized biotin-Hpep conjugates were immobilized onto a streptavidin biosensor surface. During the association step, the sensor was immersed in solutions containing labeled cpHaloΔ3, followed by immersion in buffer during the dissociation step. (**f**), Binding kinetics of TMR- or SiR-labeled cpHaloΔ3 to Hpep9 or Hpep11. The corresponding cpHaloΔ3 concentrations for each curve are annotated below the graph. Raw data and the fitted curves are represented by solid and dashed lines, respectively.

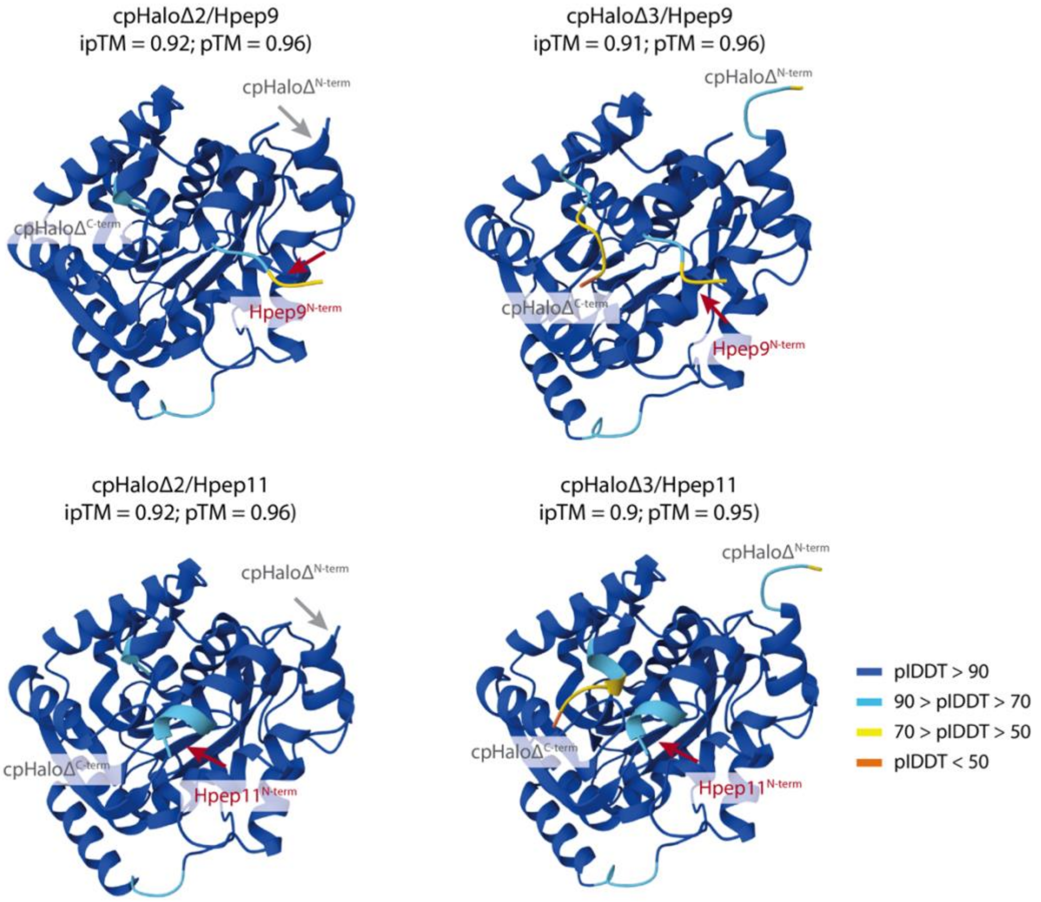

Supplementary Fig 8. Split-HaloTag structures predicted by AlphaFold3.

cpHaloΔ/Hpep complex structures were predicted using AlphaFold-Multimer with default parameters. Five models were generated per complex. The interface predicted template modeling (ipTM) and the predicted template modeling (pTM) scores of the top ranked model for each split-HaloTag pair were listed. For visualization, the predicted structures were colored according to the per-atom plDDT values, reflecting the model's confidence in the local structure. Due to the low structural confidence of Hpep9 in complex with both cpHaloΔ2 and cpHaloΔ3, particularly in the N-terminal region of the predicted α-helix, we conducted MD simulations only for the Hpep11 complex.

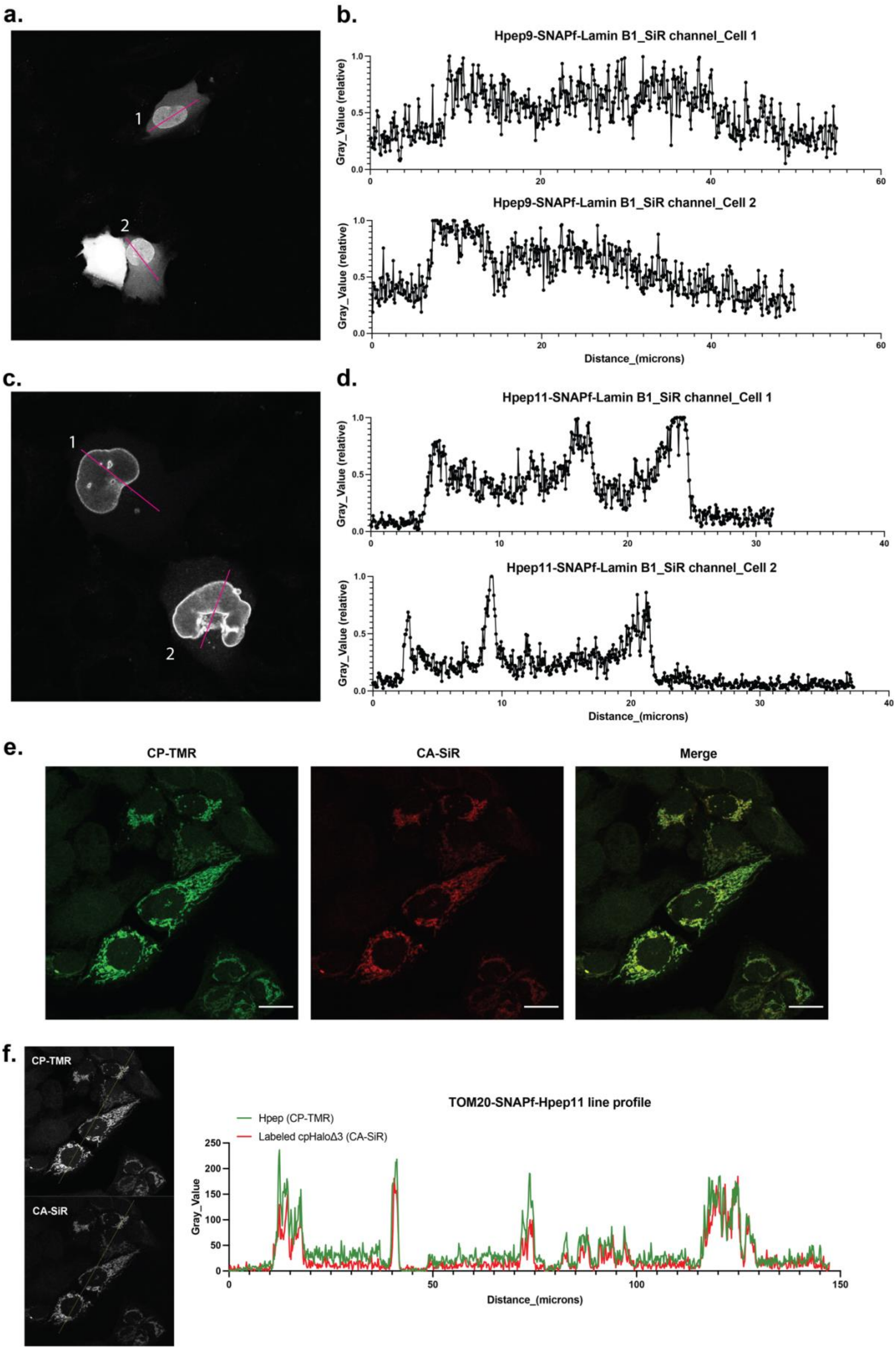
a.
1
2
b.
Hpep9-SNAPf-Lamin B1_SiR channel_Cell 1
Gray_Value (relative)
Hpep9-SNAPf-Lamin B1_SiR channel_Cell 2
Distance_(microns)
c.
d.
Hpep11-SNAPf-Lamin B1_SiR channel_Cell 1
Hpep11-SNAPf-Lamin B1_SiR channel_Cell 2
e.
CP-TMR
CA-SiR
Merge
f.
TOM20-SNAPf-Hpep11 line profile
Hpep (CP-TMR)
Labeled cpHaloΔ3 (CA-SiR)
Gray_Value

Supplementary Fig 9. Intensity profiles across cells expressing Hpep-tagged POIs.

(a-d), Confocal images and corresponding intensity profiles showing the higher signal-to-background ratio (SBR) achieved by Hpep11 compared to Hpep9 for Lamin B1 tagging in live-cell labeling.
(a), Confocal image (same as in Figure 2a, CA-SiR labeling channel of the U2OS cells co-expressing EGFP-cpHaloΔ3 and Hpep9-fused Lamin B1) shown using a grayscale LUT to highlight intensity distribution. **(b),** Intensity profiles along the lines for the two cells shown in a. **(c),** Image identical to the CA-SiR channel of Figure 2b, displayed in grayscale. **(d),** Intensity profiles along the lines for the two cells shown in c.
(e), Identical post-fixation images of Hpep11-fused TOM20 (as in Figure 2c), showing colocalization of CP-TMR (green) and CA-SiR (red), and merged channels. **(f),** The overlaid intensity profiles of CP-TMR and CA-SiR fluorescence signals show strong correlation, confirming specific labeling of Hpep11-tagged TOM20 by post-fixation staining with recombinant cpHaloΔ3 and CA-SiR.

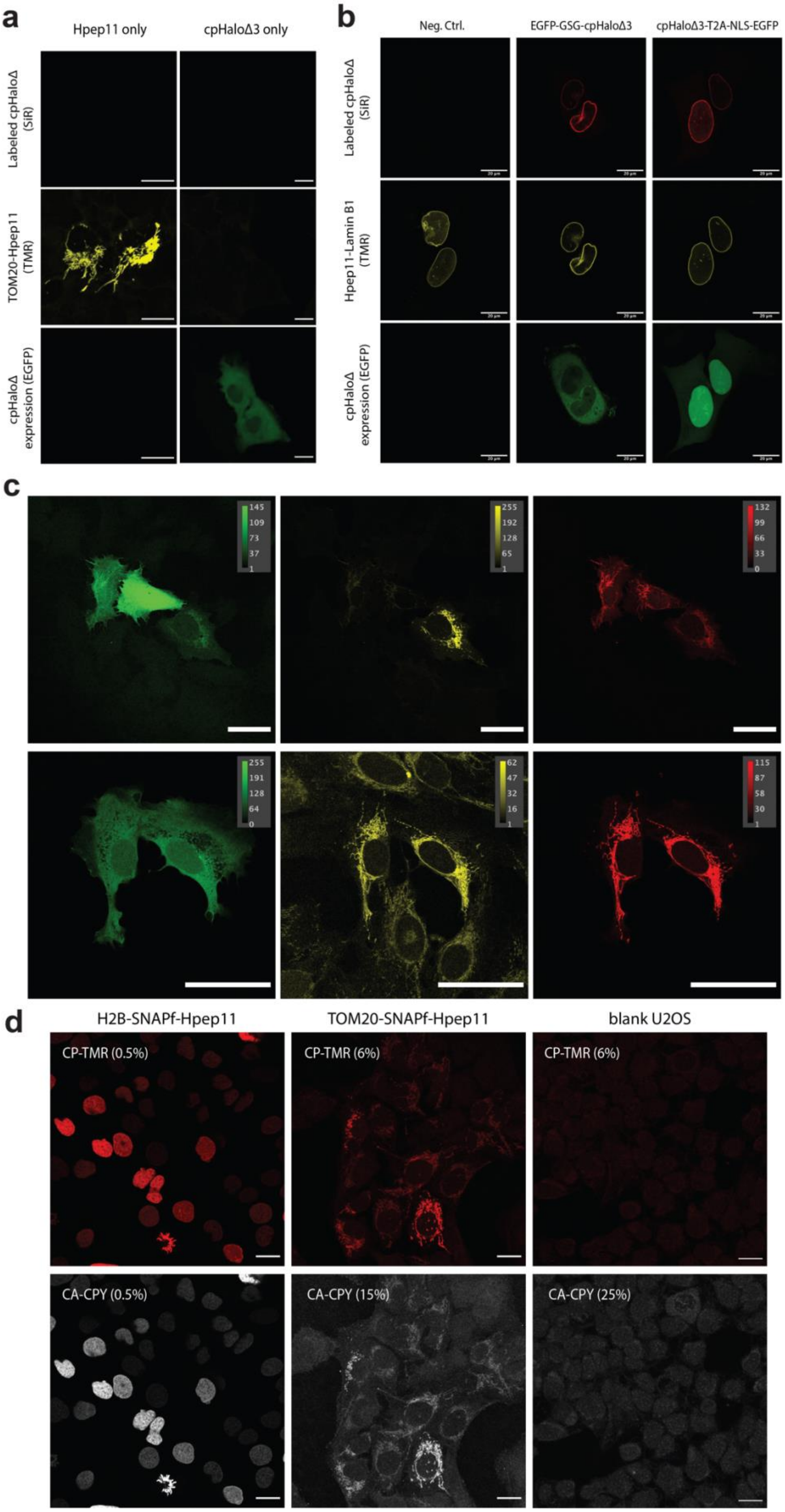
a
Hpep11 only
cpHaloΔ3 only
Labeled cpHaloΔ (SiR)
TOM20-Hpep11 (TMR)
cpHaloΔ expression (EGFP)
b
Neg. Ctrl.
EGFP-GSG-cpHaloΔ3
cpHaloΔ3-T2A-NLS-EGFP
Labeled cpHaloΔ (SiR)
Hpep11-Lamin B1 (TMR)
cpHaloΔ expression (EGFP)
c
d
H2B-SNAPf-Hpep11
TOM20-SNAPf-Hpep11
blank U2OS
CP-TMR (0.5%)
CP-TMR (6%)
CP-TMR (6%)
CA-CPY (0.5%)
CA-CPY (15%)
CA-CPY (25%)

Supplementary Fig 10. Performance of split-HaloTag in mammalian cells.

(**a**), Live-cell confocal images of U2OS cells expressing either only Hpep11 (TOM20-SNAPf-Hpep11) or cpHaloΔ3 (EGFP-GSG-cpHaloΔ3), after labeling with CP-TMR and CA-SiR. Images were taken with same laser settings. Scale bar: 20 μm. Representative images were obtained from at least five independent biological experiments, with more than 10 cells analyzed per replicate. (**b**), A comparison of cpHaloΔ3 performance in live-cell confocal imaging, with and without direct fusion to EGFP. cpHaloΔ3 with and without direct fusion to EGFP spontaneously localized to the nuclear envelope where Hpep11 was localized and resulted in a high contrast labeling in the SiR channel. Representative images were obtained from at least two independent biological experiments, with more than 10 cells analyzed per replicate. (**c**), Comparison of Hpep11 for protein visualization in live (upper panel) and fixed cells (lower panel). Confocal images of U2OS cell co-expressing EGFP-GSG-cpHaloΔ3 (stable) and TOM20-SNAPf-Hpep9 (transient transfection), labeled with 250 nM CP-TMR and 100 nM CA-CPY for 1 hour before imaging or fixation. Scale bar: 50 μm. (**d**), Post-fixation staining of Hpep11-tagged targets (H2B and TOM20) with recombinant cpHaloΔ3 [1 μM] and CA-CPY [500 nM] for overnight incubation at 4 °C. The SNAPf substrate CP-TMR [500 nM] was also included. Scale bar: 20 μm. Representative images were obtained from two independent biological experiments, analyzing over five fields of view per replicate.

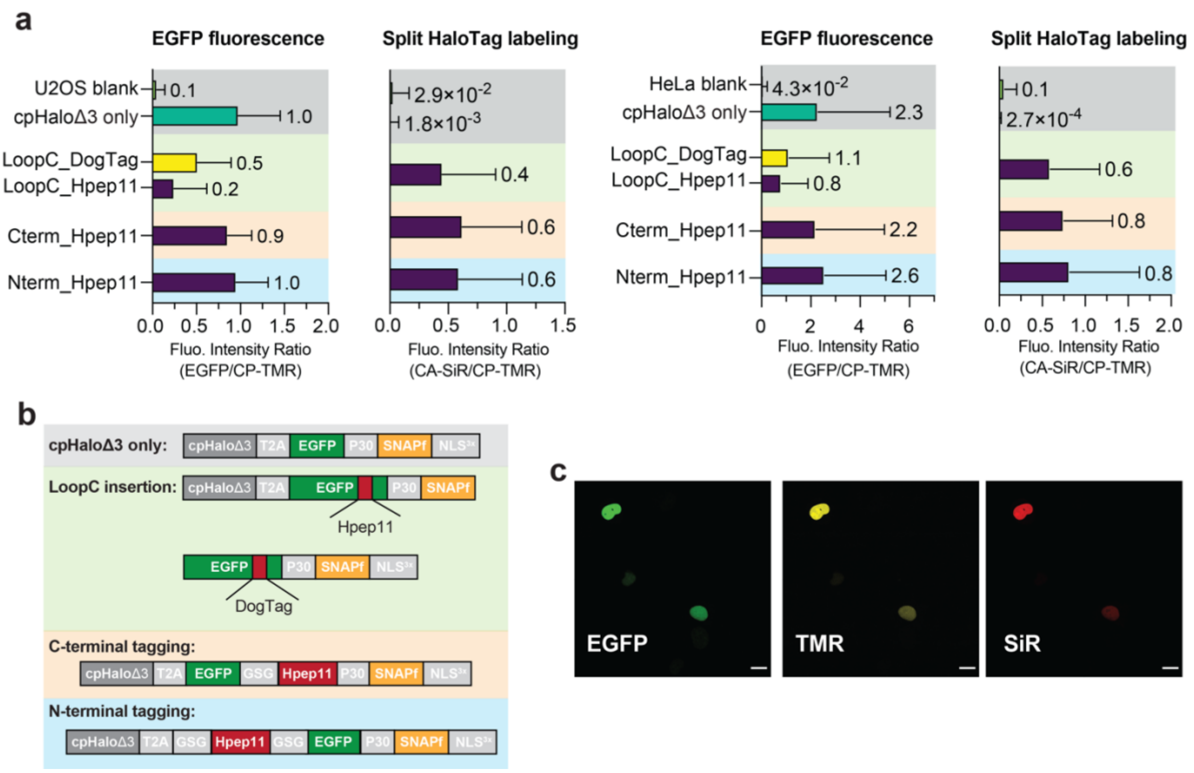

Supplementary Fig 11. Assessment of Hpep11 performance at different insertion sites in EGFP.

Performance of Hpep11 at different insertion sites of EGFP in mammalian cells, evaluated by EGFP brightness and cpHaloΔ3 labeling intensity. (**a**), Flowcytometry analysis of different tags (Hpep11, DogTag[3]) inserted either at each terminus or loopC[3] of EGFP. U2OS (left two panels) and HeLa (right two panels) cells transiently transfected with the indicated constructs were labeled with the SNAPf substrate CP-TMR to normalize expression levels. For split HaloTag labeling measurements, cells were additionally labeled with CA-SiR. The mean and standard error of the mean (SEM) of each cell population with TMR^{+} signal are shown here. Sample sizes (n), referring to the number of gated TMR+ events precondition, are provided in the Source Data Excel sheet. (**b**), The corresponding constructs for the analysis shown in (a). The P30 linker was designed to minimize fluorescence resonance energy transfer (FRET) between EGFP and CP-TMR, ensuring accurate expression normalization based on the TMR signal. (**c**), Representative confocal live-cell images (n=10~20 cells from one biological experiment) of U2OS cells transfected with the construct of Hpep11 inserted in loopC of EGFP. After labeling with CP-TMR and CA-SiR, the correct subcellular localization of fluorescence signal in each channel was confirmed. Scale bar: 20 μm.

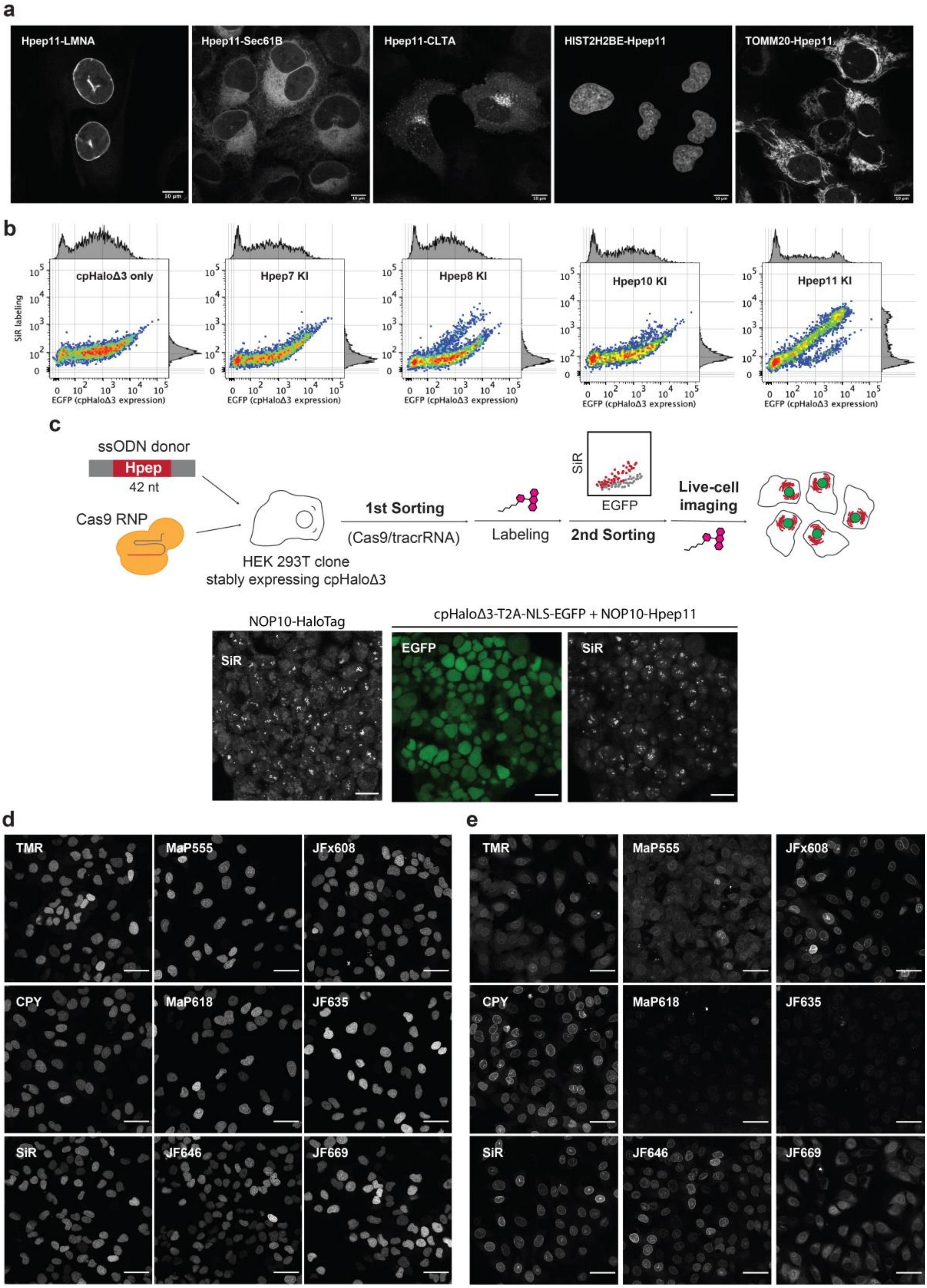

Supplementary Fig 12. The performance of Hpep variants for endogenous protein labeling.

(**a**), Representative live-cell confocal images (n≥3 biological independent experiments, with >20 cells imaged in each experiment) of the Hpep11 CRISPR KI cell lines after labeling with 100 nM CA-SiR. (**b**), Flow cytometry analysis of the labeling performance of Hpep variants that were knocked in at *TOMM20* in the cpHaloΔ3 (cpHaloΔ3-T2A-NLS-EGFP) overexpressing cell line, after labeling with CA-SiR [100 nM, 1 hour]. (**c**), Workflow for the generation of Hpep11 CRISPR cell line in human embryonic kidney (HEK) 293FT cells with cpHaloΔ3-expressing cassette (cpHaloΔ3-T2A-NLS-EGFP) stably integrated into safe harbour AAVS1 locus (top). The illustration was created using Adobe Illustrator with some elements adapted from BioRender.com. Live-cell imaging of HaloTag- or Hpep11-tagged cell lines after one-hour labeling of CA-SiR [100 nM] without wash. Scale bar: 20 μm. Representative images from 2 biological independent experiments. (**d-e**), Live-cell confocal imaging of histone H2B type-2E-Hpep11 (d.) or Hpep11-lamin A/C (e.) CRISPR KI cell lines after 2-hours labeling with the specified CA- ligand [100 nM]. Images were taken with optimal image acquisition parameter for each dye. Scale bar: 50 μm. Images were acquired from a single experiment, with at least three fields of view analyzed for each staining condition.

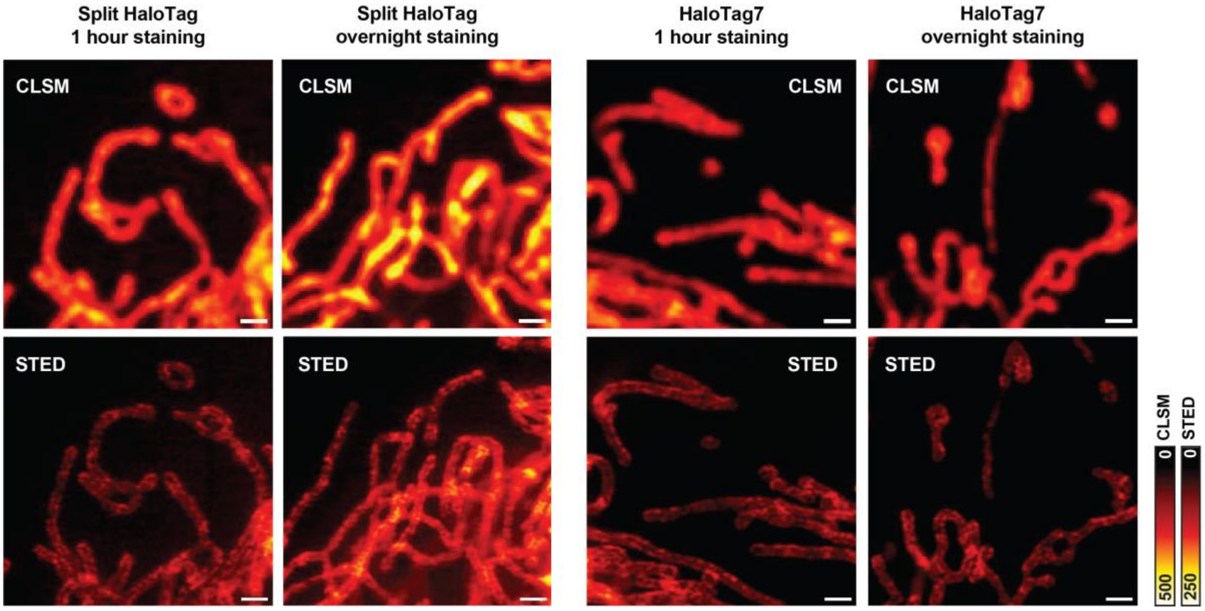

Supplementary Fig 13. Comparison of split-HaloTag and HaloTag performance in live-cell imaging of endogenously tagged TOM20.

Live-cell CLSM and STED imaging of CRISPR KI cell lines. Hpep11-tagged TOM20 cells overexpressing cpHaloΔ3-NLS-EGFP and TOM20-HaloTag CRISPR cells were labeled with 100 nM CA-SiR for 1 h or overnight (21 h), followed by two washes prior to imaging. Under these conditions, split-HaloTag exhibited a SBR comparable to that of HaloTag. These images are representative of n=3 independent experiments, with three images acquired from each experiment. Scale bar: 1 µm. ‘Red Hot’ LUT and Gaussian blur (1 pixel) applied.

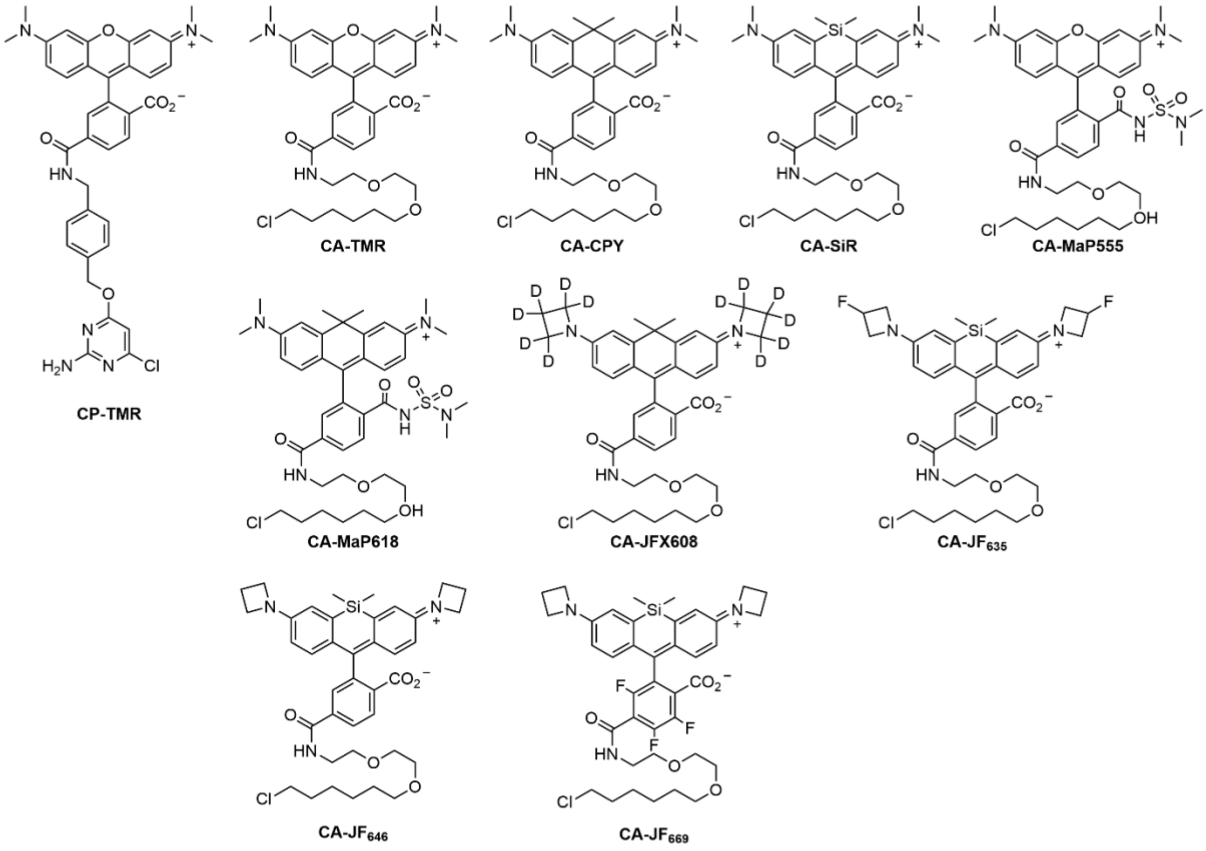

Supplementary Fig 14. Fluorescent SLP substrates used in this study.

Fluorophore-coupled SNAP-tag (CP-) and HaloTag (CA-) substrates.

a

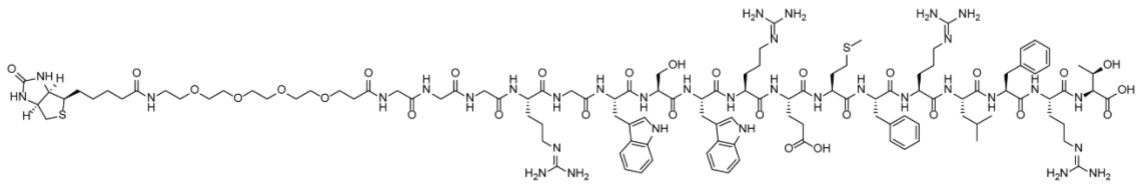

b

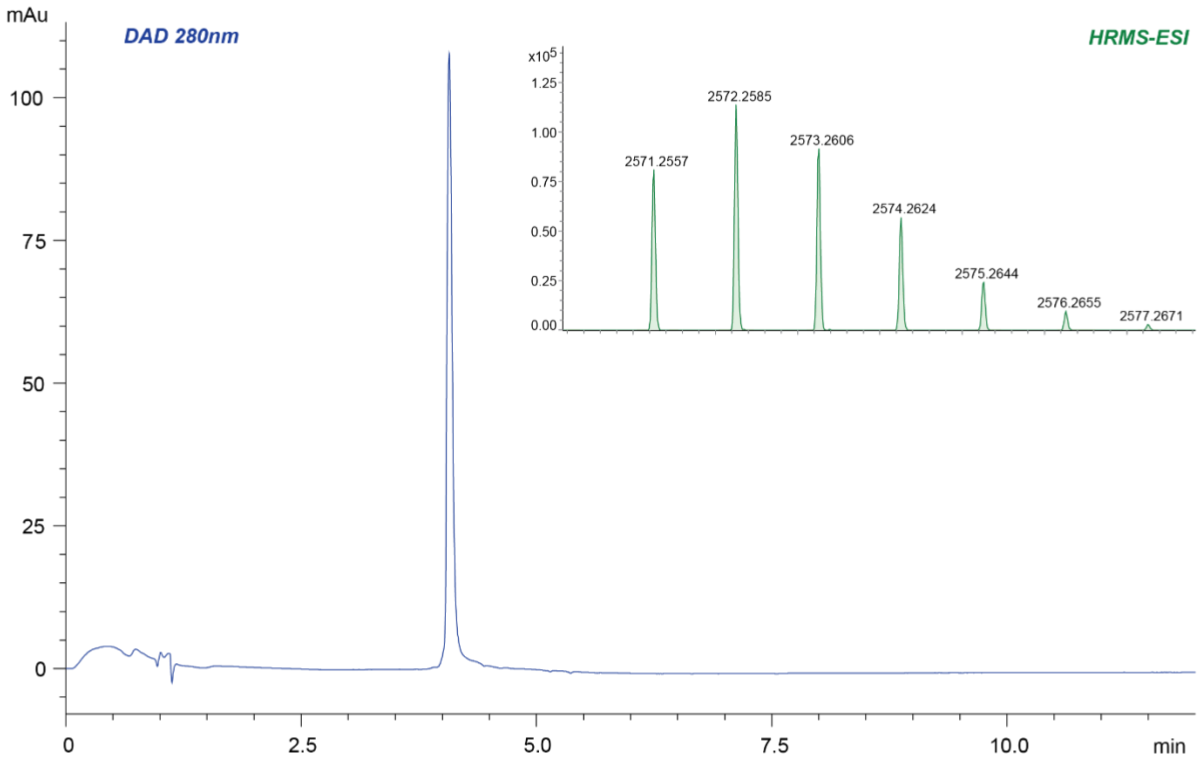

Supplementary Fig 15. HPLC-HRMS analysis of the biotinylated Hpep9.

(**a**), Chemical structures of biotinylated Hpep9. (**b**), HPLC-MS analysis of the purified peptide, showing the 280 nm DAD chromatogram and the isotopically resolved intact mass of the corresponding DAD peak. High-resolution mass spectrometry (HRMS, ESI) analysis: calculated mass for $C_{116}H_{174}N_{34}O_{29}S_2$, 2571.2627; observed mass, 2571.2557.

a

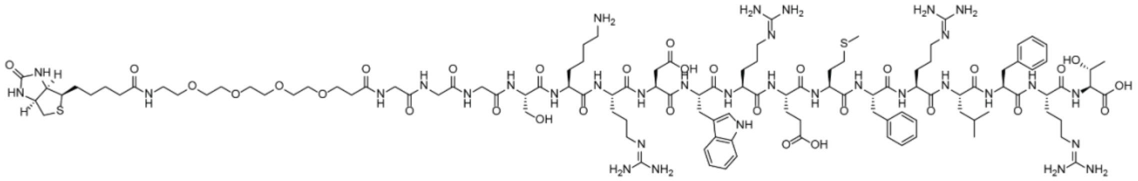

b

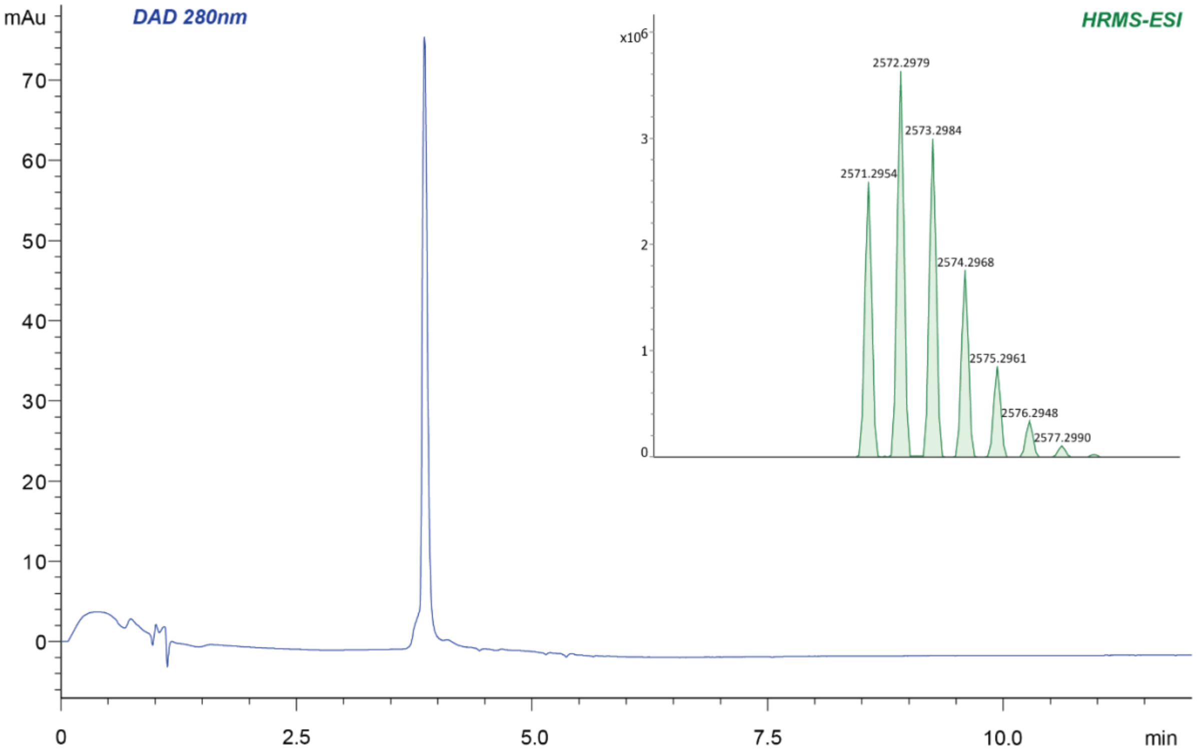

Supplementary Fig 16. HPLC-HRMS analysis of the biotinylated Hpep11.

(**a**), Chemical structures of biotinylated Hpep11. (**b**), HPLC-MS analysis of the purified peptide, showing the 280 nm DAD chromatogram and the isotopically resolved intact mass of the corresponding DAD peak. HRMS (ESI) analysis: calculated mass for $C_{113}H_{178}N_{34}O_{31}S_2$, 2571.2839; observed mass, 2571.2954.

a

b

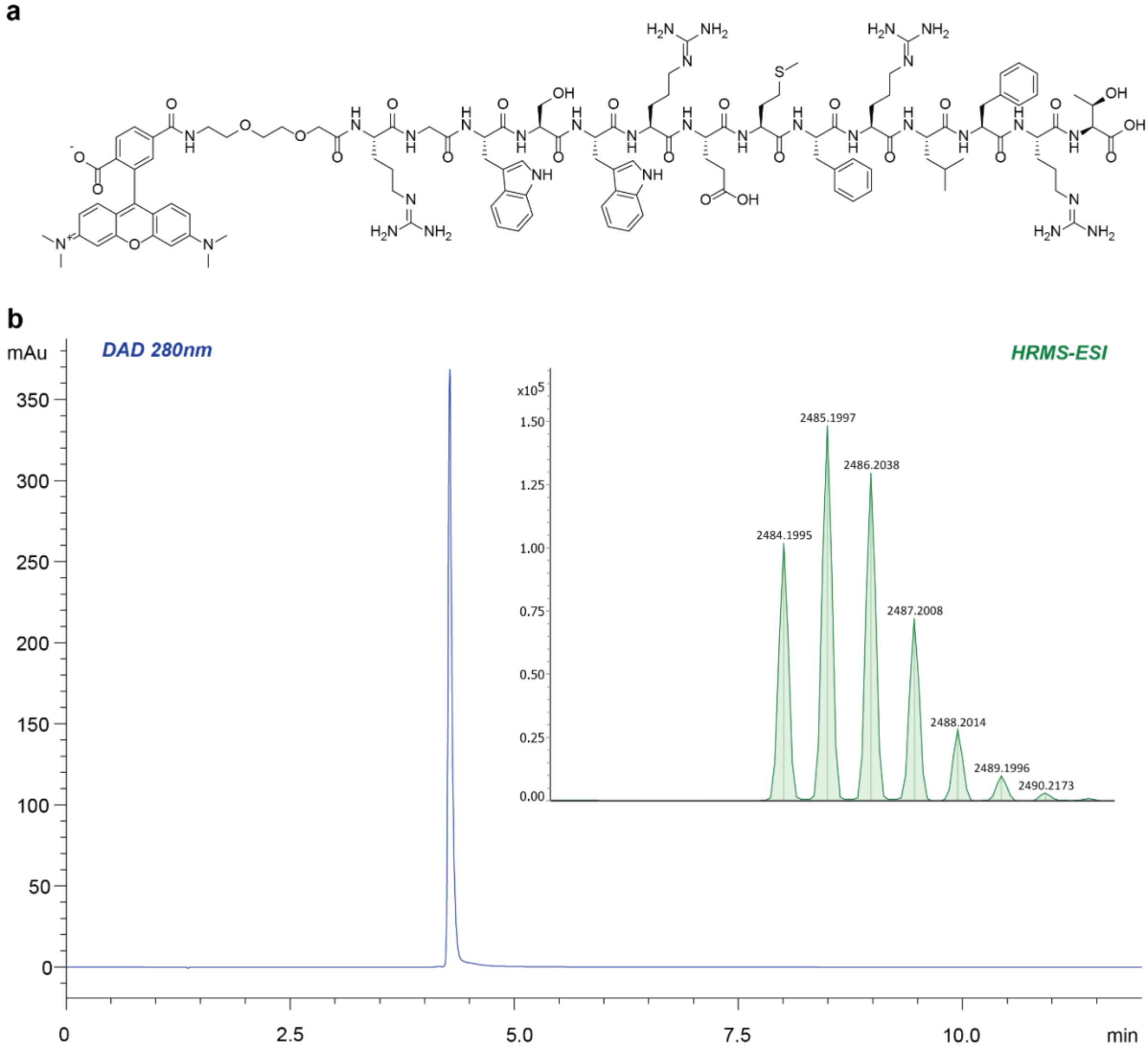

Supplementary Fig 17. HPLC-HRMS analysis of the TMR-conjugated Hpep9.

(**a**), Chemical structures of TMR-labeled Hpep9. (**b**), HPLC-MS analysis of the purified peptide, showing the 280 nm DAD chromatogram and the isotopically resolved intact mass of the corresponding DAD peak. HRMS (ESI) analysis: calculated mass for $C_{120}H_{161}N_{31}O_{26}S$, 2484.1950; observed mass, 2484.1995.

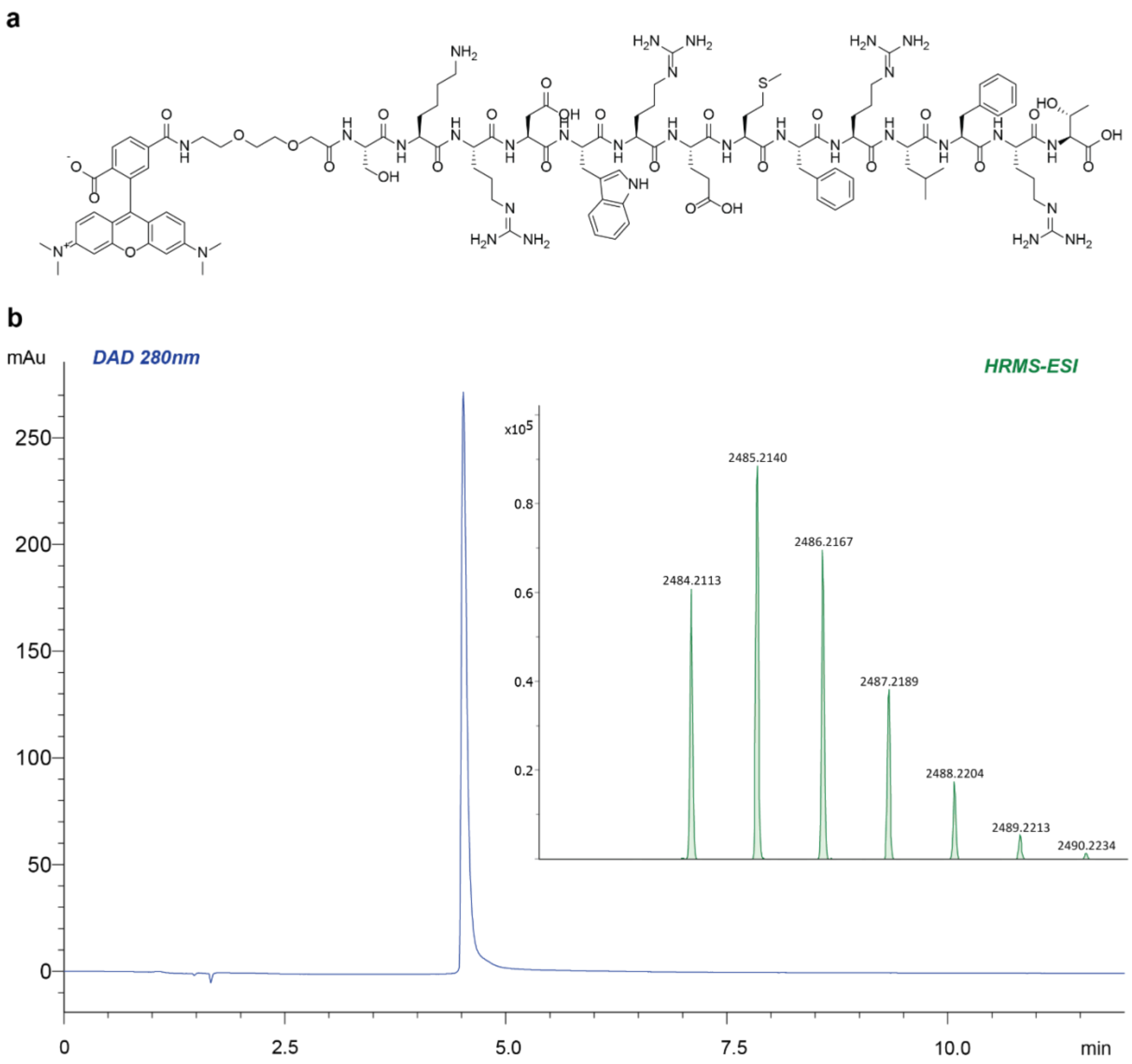

Supplementary Fig 18. HPLC-HRMS analysis of the TMR-conjugated Hpep11.

(**a**), Chemical structures of TMR-labeled Hpep11. (**b**), HPLC-MS analysis of the purified peptide, showing the 280 nm DAD chromatogram and the isotopically resolved intact mass of the corresponding DAD peak. HRMS (ESI) analysis: calculated mass for $C_{117}H_{165}N_{31}O_{28}S$, 2484.2161; observed mass, 2484.2113.

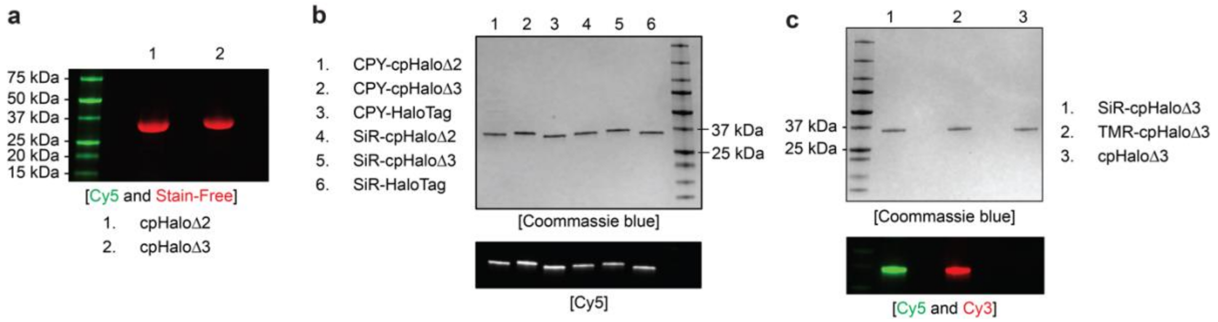

Supplementary Fig 19. SDS-PAGE analysis of the recombinant cpHaloΔ proteins.

The size and the labelling completeness of the cpHaloΔ proteins were verified by SDS-PAGE. The expected sizes for cpHaloΔ2 and cpHaloΔ3 is 36.4 kDa and 37.3 kDa, respectively. (**a**), Verification of the purify of cpHaloΔ proteins following His-tag purification, assessed using Stain-Free^{TM} precast PAGE gels. (**b**), Verification of the fluorescently-labeled cpHaloΔ prepared for fluorescence emission scan assay (Fig.1f and Supplementary Fig 6). (**c**), Verification of the fluorescently-labeled cpHaloΔ3 prepared for FP and BLI measurements (Supplementary Fig 7). Fluorescence signal of SiR and CPY was collected in Cy5 channel, while TMR was collected in the Cy3 channel.

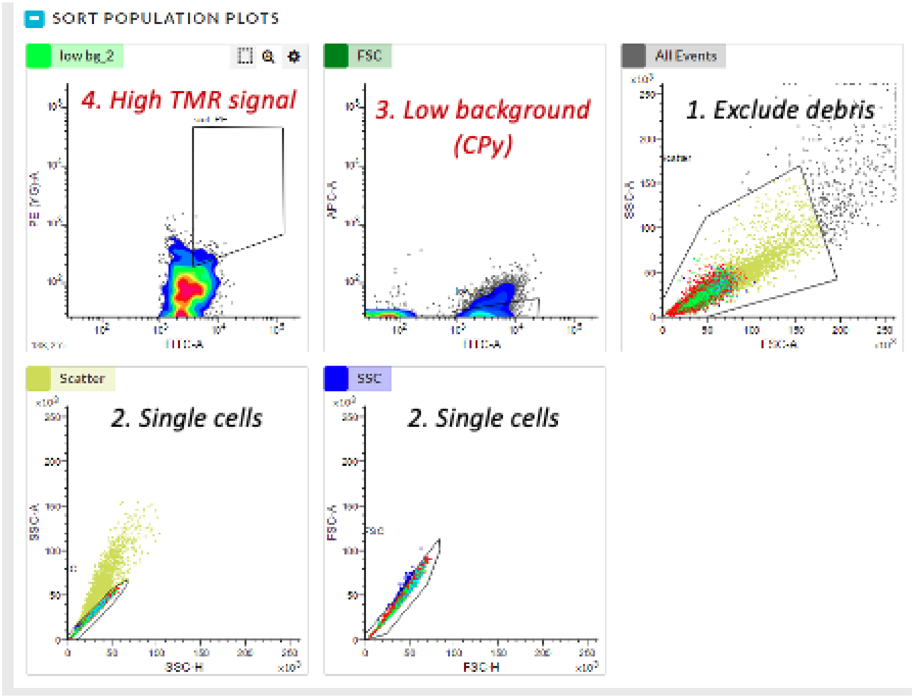

Supplementary Fig 20. Gating strategy for yeast library screening.

Living yeast cells were gated first using SSC-A/FSC-A, followed by singlet gating using FSC-A/FSC-H. Within the singlet population, yeast cells showing low CPY-labeling signal represented those with low residual labeling activity in absence of Hpep were gated. Within this population, the yeast cells showing high TMR signal were then sorted.

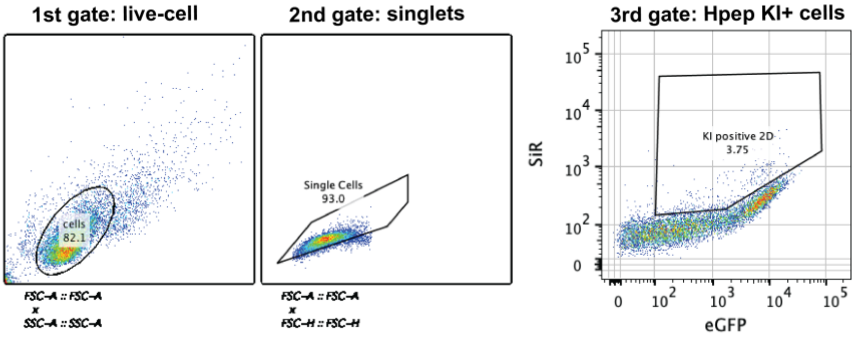

Supplementary Fig 21. Gating strategy to enrich Hpep11-integrated cells.

Cells were labeled with 100 nM CA-SiR for one hour. Hpep KI-positive cells co-expressing cpHaloΔ3-T2A-NLS-EGFP were enriched by FACS using the representative gating strategy.

Supplementary Tables

Supplementary Table 1. EC_{50} values of Hpep variants for the parental cpHaloΔ.

Hpep	Sequence	EC_{50} (μM)	95% confidence interval
Hpep1[a]	ARETFQAFRT	2979	2628 – 3417
Hpep2[a]	AREMFQAFRT	1116	928 – 1350
Hpep3[a]	SKRDAREMFQAFRT	149	128 – 176
Hpep4[a]	WKEEVIKAFKLFRD	21.0	18.3 -24.1
Hpep5[a]	WREEVRKAFKLFRQ	10.7	8.84 – 13.0
Hpep6[a]	WRETFQLFRT	2.39	2.08 – 2.77
Hpep7[a]	WREMFRLFRT	0.35	0.32 – 0.38
Hpep8[a]	WKRDWREMFRLFRT	0.12	0.11 – 0.14
Hpep9[b]	RGWSWREMFRLFRT	0.040	0.031 – 0.051
Hpep10[b]	RMWTWREMFRLFRT	0.047	0.030 – 0.070
Hpep11[b]	SKRDWREMFRLFRT	0.235	0.225 – 0.245

[a]The EC_{50} values for Hpep1-Hpep8 are sourced from the previous publication from our group[4], and [b]the values for Hpep9-Hpep11 are reported in the PhD thesis of Jonas Wilhelm. The EC_{50} characterization via CA-TMR labeling kinetics assays were done with TEVp-cleaved cpHaloΔ protein, followed by reverse-IMAC and SEC purification. For Hpeps with EC_{50} values >1 μM, final assay concentrations of 500 nM cpHaloΔ protein and 100 nM CA-TMR substrate were used. For Hpeps with EC_{50} values <1 μM, the assays were performed with 20 nM cpHaloΔ protein and 4 nM CA-TMR. Hpeps were titrated over a concentration range from 0 to 5 mM, depending on the EC_{50} of the respective Hpep.

Supplementary Table 2. Affinity measurement of labeled-cpHaloΔ3 to biotin-Hpep conjugates.

Sample	K_d (M)	K_d error	k_{on} (1/Ms)	k_{on} error	k_{off} (1/s)	k_{off} error	Full R^2
Hpep11/ cpHaloΔ3-SiR	4.27E-09	2.29E-11	5.00E+04	6.55E+01	2.14E-04	1.11E-06	0.9995
Hpep9/ cpHaloΔ3-SiR	1.88E-08	1.57E-10	7.89E+04	6.17E+02	1.49E-03	4.24E-06	0.996
Hpep11/ cpHaloΔ3-TMR	1.06E-08	2.75E-11	6.49E+04	8.97E+01	6.87E-04	1.51E-06	0.9993
Hpep9/ cpHaloΔ3-TMR	4.86E-09	6.15E-11	2.14E+05	2.30E+03	1.04E-03	1.04E-06	0.9899

The K_d, k_{on}, and k_{off} error values represent the standard error of the mean (S.E.M). The full R^2 values were calculated from the global fit to the 1:1 binding model.

Supplementary Table 3. CRISPR/Cas9 KI target information.

Target gene name	Target protein_name	Hpep-tag location	crRNA_sequence (5' – 3')	HDR_donor_sequence<br>lower case: homology arms; red: Hpep11; blue: linker	RNA expression in U2OS (nTPM)	Localization/ subcellular structure
VIM	Vimentin	N-terminus	GGACCTGGTGGACATGGCTG	**Hpep5:**cgttcgcctcttctccgggagccagtccgcgccaccgccgccgcccaggccatcgccaccctccgcagccATGAGTAAACGCGACTGGAGGGAGATGTTTAGGCTTTTTCGGACAGGTGGCGGCtccaccaggtccgtgtcctcgtcctcctaccgcaggatgttcggcggcccgggcaccgcgagccggccga	1492.8	Intermediate filament
TUBB4B	Tubulin beta-4B chain	N-terminus	CGCCGCCGCCGCCATCATGA	**Hpep5:**ctgctgctgtttgtctacttcctcctgcttccccgccgccgccgccgccatcATGAGTAAACGCGACTGGAGGGAGATGTTTAGGCTTTTTCGGACAGGTGGCGGCagggaaatcgtgcacttgcaggccgggcagtgcggcaaccaaatcggcgccaagg	793.1	Microtubules
LMNA	Prelamin-A/C	N-terminus	CCATGGAGACCCCGTCCCAG	**Hpep5:**tccttcgacccgagccccgcgccctttccgggacccctgccccgcgggcagcgctgccaacctgccggccATGAGTAAACGCGACTGGAGGGAGATGTTTAGGCTTTTTCGGACAGGTGGCGGCgagaccccgtcccagcggcgcgccacccgcagcggggcgcaggccagctccactccgctgtcgcccaccc	255	Nuclear speckles
SEC61B	Protein transport protein Sec61 subunit beta	N-terminus	GCTTGTCTCCCTCTACAGCC	**Hpep5:**gtgtctaggccggggttctggggcaggcctgccgcgctcacccgtctgtctgcttgtctccctctacagATGAGTAAACGCGACTGGAGGGAGATGTTTAGGCTTTTTCGGACAGGTGGCGGCcctggtccgacccccagtggcactaacgtgggatcctcagggcgctctcccagcaaagcagtggccgcccgggc	248	Endoplasmic reticulum
CLTA	Clathrin light chain A	N-terminus	AGCCATGGCGGGCAACTGAA	**Hpep5:**cgggcgtggtgtcggtgggtcggttggtttttgtctcaccgttggtgtccgtgccgttcagttgcccgccATGAGTAAACGCGACTGGAGGGAGATGTTTAGGCTTTTTCGGACAGGTGGCGGCgctgagctggatccgttcggcgcccctgccggcgcccctggcggtcccgcgctggggaacgga	215	Vesicles, endosomes, lysosomes
TOMM20	Mitochondrial import receptor subunit TOM20	C-terminus	GAGCTTGGCTGAAGATGATG	**Hpep5:**cagagaattgtaagtgctcagagcttagctgaagatgatgttgaaGGTGGCGGCAGTAAACGCGACTGGAGGGAGATGTTTAGGCTTTTTCGGACATGAgaaacaaatgtcaacataataaaatctcagtt<br>**Hpep4:**cagagaattgtaagtgctcagagctt**a**gctgaagatgatgt**t**gaaGGTGGCGGCAGAGGATGGTCATGGCGCGAAATGTTTCGGCTTTTTCGGACGTGAgaaacaaatgtcaacataataaaatctcagtt	54.8	Mitochondria
HIT2H2BE /H2BC21	Histone H2B type 2-E	C-terminus	GGTCCCGGCAGGGACTCACT	**Hpep5:**gcccggcgagctggccaagcacgccgtgtccgagggcaccaaggcggtcaccaagtacaccagctccaagGGTGGCGGCAGTAAACGCGACTGGAGGGAGATGTTTAGGCTTTTTCGGACAtgagtccctgccgggacctggcgctcgctcgctcgagtcgccggctgcttgactccaaaggctcttttcagag	32.2	Nucleoplasm

The information was sourced from the Human Protein Atlas[5]

Supplementary Table 4. Fluorescence lifetime of split-HaloTag pairs.

Split-HaloTag pair	lifetime (ns)	S.E.M	N (cells)
cpHaloΔ3_cytosol	2.285	0.0927	5
cpHaloΔ3_nuclus	2.477	0.0190	5
cpHaloΔ3/Hppe8	3.07	0.017	5
cpHaloΔ3/Hppe9	2.63	0.044	7
cpHaloΔ3/Hppe10	2.22	0.024	7
cpHaloΔ3/Hppe11	3.52	0.033	10

Lifetime values represent the mean measurement over the N number of cells indicated. S.E.M. = standard error of the mean.

Supplementary Table 5. Medium and buffers used in this study.

Medium / Buffer	Composition
LB medium (agar)	10 g/L tryptone, 5 g/L yeast extract, 10 g/L NaCl, pH 7.5 (15 g/L agar)
Activity buffer (10×)	500 mM HEPES, 500 mM NaCl, pH 7.3
Gibson assembly enzyme mix	0.64 μL T5 exonuclease (10 U/μL), 20 μL Phusion polymerase (2 U/μL), 160 μL Taq ligase (40 U/μL), 320 μL ISO buffer (5×), 700 μL H_2O, aliquoted to 20 μL/reaction
His extract buffer	50 mM KH_2PO_4, 300 mM NaCl, 5 mM imidazole, pH 8.0
His washing buffer	50 mM KH_2PO_4, 300 mM NaCl, 10 mM imidazole, pH 7.5
His elution buffer	50 mM KH_2PO_4, 300 mM NaCl, 500 mM imidazole, pH 7.5
TEVp buffer	25 mM Tris-HCl, 200 mM NaCl, 1 mM DTT, 2.5% glycerol, pH 8.0
Sample buffer (4x)	1:9 mixture of 2-mercaptoethanol and 4x Laemmli sample buffer (Bio-Rad)
TAE buffer (50x)	2 M Tris, 50 mM EDTA (pH8.0), 1 M glacial acetic acid
YPD medium (agar)	20 g/L tryptone, 10 g/L yeast extract, 2% glucose (15 g/L agar)
YPDS medium	1:1 mixture of YPD medium and 1 M sorbitol
SDCAA medium (agar)	6.7 g/L yeast nitrogen base, 5 g/L yeast synthetic drop-out medium supplement without tryptophan, 13.6 g/L $Na_2HPO_4 \cdot 12H_2O$, 8.56 g/L $NaH_2PO_4 \cdot H_2O$, 2% glucose (15 g/L agar)
SGCAA medium	6.7 g/L yeast nitrogen base, 5 g/L yeast synthetic drop-out medium supplement without tryptophan, 13.6 g/L $Na_2HPO_4 \cdot 12H_2O$, 8.56 g/L $NaH_2PO_4 \cdot H_2O$, 2% galactose
Amino acid supplement solution	6.7 g/L yeast nitrogen base without amino acids, 5 g/L yeast synthetic drop-out medium supplement without tryptophan
Electroporation buffer	10 mM Tris-base, 0.27 M sucrose, 2.1 mM $MgCl_2$, pH 7.5
DTT-Tris buffer	0.39g dithiothreitol dissolved in 1 mL of 1 M Tris-HCl, pH 8.0
LiAc-Tris buffer	1g lithium acetate dissolved in 2 mL of 1 M Tris-HCl, pH 8.0
Cell growth medium	DMEM high glucose, 10% FBS
Imaging medium	High-glucose phenol-red free DMEM medium, 4.5 g/L glucose, 110 mg/L pyruvate, 1x GlutaMAX™, 10% FBS
FACS buffer	2% FBS in PBS (Thermo Fisher Scientific)
U-ExM monomer solution	19% (w/w) sodium acrylate (SA), 10 % (w/w) AA, 0.1% (w/w) N,N'-methylenebisacrylamide (BIS) in PBS
4% PFA	Diluted from 16% PFA with PBS
Denaturation buffer	200 mM sodium dodecyl sulfate (SDS), 200 mM NaCl and 50 mM Tris, pH 9.0

Supplementary Table 6. Spectral properties, reactive protein tags and full names of the fluorescent ligands used in this study.

Fluorescent ligands	Reactive protein tag	Full name/ Commercial name from the vendor	Abs/Em [nm]
CP-TMR	SNAP-tag and SNAPf	Chloropyrimidine (CP) derivative of tetramethylrhodamine fluorophore / SNAP-Cell TMR-Star (New England Biolabs)	554/580
CA-TMR	HaloTag7 and split-HaloTag (cpHaloΔ fragment)	Chloroalkane (CA) derivative of tetramethylrhodamine fluorophore	555/580
CA-MaP555[6]	HaloTag7 and split-HaloTag (cpHaloΔ fragment)	- / SPY555-CA (Spirochrome)	555/580
CA-JFX608[7]	HaloTag7 and split-HaloTag (cpHaloΔ fragment)	- / JFX_{608}–HaloTag	608/628
CA-CPY	HaloTag7 and split-HaloTag (cpHaloΔ fragment)	CA derivative of the fluorophore carbopyronin / CPY-CA (Spirochrome)	606/626
CA-MaP618	HaloTag7 and split-HaloTag (cpHaloΔ fragment)	- / MaP618–CA	618/635
CA-JF635[8]	HaloTag7 and split-HaloTag (cpHaloΔ fragment)	CA derivative of the Janelia Fluor 635 / -	635/652
CA-SiR	HaloTag7 and split-HaloTag (cpHaloΔ fragment)	CA derivative of the silicon rhodamine (SiR) fluorophore / SiR-CA (SPIROCHROME)	652/674
CA-JF646[8]	HaloTag7 and split-HaloTag (cpHaloΔ fragment)	CA derivative of the Janelia Fluor 646 / -	646/664
CA-JF669[7]	HaloTag7 and split-HaloTag (cpHaloΔ fragment)	CA derivative of the Janelia Fluor JFX608 / -	669/682

Supplementary Table 7. Spectral properties of fluorophores used in this work.

Fluorescent substrate	λ_{abs} [nm]	Buffer	ε [$M^{-1}·cm^{-1}$]
TMR[9]	555	PBS	89,000
MaP555[10]	558	0.1% SDS in PBS	142,000
JFx608[11]	608	10 mM HEPES, pH 7.3	111,000
CPY[12]	616	0.1% SDS in PBS	152,000
MaP618[10]	616	0.1% SDS in PBS	5,500
JF635[8]	635	0.1% SDS in PBS	17,000
SiR[13]	646	0.1% SDS in PBS	120,000
JF646[14]	646	0.1% SDS in PBS	106,000
JF669*	674	0.1% SDS in PBS	128,500

Extinction coefficients were extracted from literature and/or measured in-house* in the provided buffer composition.

Supplementary Table 8. PCR reaction recipe for cpHaloΔ library generation.

Reagent	Volume (µL)
KOD polymerase master mix	50
H_2O	46
Forward Primer 10 µM	1.5
Reverse Primer 10 µM	1.5
DNA template at 2 ngµL^{-1}	1.0

Supplementary Table 9. PCR reaction protocol for cpHaloΔ library generation.

Step	Temperature (°C)	Duration(s)	Cycles
Initial denaturation	95	120	1x
Denaturation	95	30	
Primer annealing	68	20	35x
DNA synthesis (elongation)	70	210	
Final elongation	70	300	1x

Supplementary Table 10. Digestion reaction recipe for cpHaloΔ library generation.

Reagent	Volume (μL)
DNA (PCR product)	60
H_2O	9.5
rCutSmart buffer	8
NcoI-HF or NotI-HF	2.5

Supplementary Table 11. Ligation reaction recipe for cpHaloΔ library generation.

Reagent	Volume (μL)
T4 DNA ligase buffer	10
H_2O	77
DNA	8
T4 DNA ligase	5

Supplementary Table 12. FACS and flow cytometry analysis.

Fluorescent molecules	Excitation Laser (nm)	Emission Filter (nm)	Machine
eUnaG, EGFP	488	530/30	Fortessa
TMR	561	580/15	Fortessa
CPY	640	670/30	Fortessa
SiR	640	670/30	Fortessa
eUnaG, EGFP	488	527/32	Melody
TMR	561	582/15	Melody
CPY	640	660/10	Melody
SiR	640	660/10	Melody

Supplementary Table 13. Sorting strategy for N-terminal extension cpHaloΔ library.

Screening round	Gating	Sort count	Total events	Hpep9 concentration	Incubation time
Round 1	counter	17,915	1,706,693	2 μM	30 min
Round 2	counter	20,000	1,403,985	1 μM	30 min
Round 3	counter	10,000	985,765	500 nM	30 min
Round 4	counter	10,000	884,280	500 nM	15 min
Round 5	negative	20,000	148,213	250 nM	30 min
Round 6	counter	10,000	915,701	250 nM	30 min

Incubation time corresponds to the time of CA-TMR (1 μM) incubation. Counter gating was performed on yeast cells labeled with CA-TMR in the presence of Hpep9, while negative gating was performed on yeast cells labeled with CA-TMR in the absence of Hpep9 as illustrated in Supplementary Fig 1a.

Supplementary Table 14. Sorting strategy for C-terminal extension cpHaloΔ library.

Screening round	Gating	Sort count	Total events	Hpep9 concentration	Incubation time
Round 1	counter	16,855	1,560,303	2 μM	30 min
Round 2	counter	16,480	1,214,859	1 μM	30 min
Round 3	counter	10,000	904,603	500 nM	15 min
Round 4	counter	10,000	1,068,002	250 nM	30 min
Round 5	counter	10,000	917,019	125 nM	30 min

Supplementary Table 15. Primers for library generation and NGS sample preparation.

Library	Purpose	Forward primer sequence	Reverse primer sequence
YSD cpHaloΔ N-term	NNK library generation	**CCATGG**TAGGTTCTGGC*NNKNNKNNKNNK*GACGTCGGCCGCAAGC	**CCATGG**TACCATTAGCTGGAGCAGCC
YSD cpHaloΔ C-term	NNK library generation	**GCGGCCGC**TTTCTCCCAAAAGTTGG	**GCGGCCGC***MNNMNNMNNMNN*CCATTCGTCCCAGGTCGG
YSD cpHaloΔ N-term	NGS sample preparation	TCCCATCTATTTTCACCGCTGTTG	GCAGCGTACCCTCGATAAAAAC
YSD cpHaloΔ C-term	NGS sample preparation	GTTTCCACTGGGCCAAGC	GACAACGTTATCCAACAAGTTGATGTC

Primer sequences with **restriction enzyme digestion site**, degenerate codons, and annealing sequences specified.

Supplementary Table 16. Stable cell lines generated in this study.

Cell type	Plasmid 1	Stable integration method	Plasmid 2	Stable integration method	Figure
U2OS	pcDNA5/FRT/TO-EGFP-GSG-cpHaloΔ2	Flp-IN	-	-	Supplementary Fig 4
U2OS	pcDNA5/FRT/TO-EGFP-GSG-cpHaloΔ3	Flp-IN	-	-	Fig. 3f-g;<br>Supplementary Fig 4
U2OS	pcDNA5/FRT/TO-H2B-SNAPf-Hpep11	Flp-IN	-	-	Fig. 2c;<br>Supplementary Fig 10d
U2OS	pcDNA5/FRT/TO-TOMM20-SNAPf-Hpep11	Flp-IN	-	-	Fig. 2c;<br>Supplementary Fig 10d
U2OS	pAAVS1-P-TO-EGFP-GSG-cpHaloΔ3	AAVS1 safe harbor	pcDNA5/FRT/TO-TOMM20-SNAPf-Hpep11	Flp-IN	Fig. 2d; Fig. 3f, 3g; Fig. 4a;<br>Supplementary Fig 10c
U2OS	GGG-Hpep or Hpep-GGG	CRISPR/Cas9 KI	pcDNA5/FRT/TO- cpHaloΔ3-T2A-NLS-EGFP	Flp-IN	Fig. 3; Fig. 4; Fig. 5b;<br>Supplementary Fig 12a-c;<br>Supplementary Fig 12e
U2OS	GGG-HaloTag	CRISPR/Cas9 KI	-	-	Fig. 3
U2OS	HaloTag-GGG	CRISPR/Cas9 KI	-	-	Fig. 4d
U2OS	pcDNA5/FRT/TO-cpHaloΔ3-T2A-NLS-EGFP	Flp-IN	-	-	Fig. 3c-d;<br>Supplementary Fig 12e
293T	pAAVS1-P-TO_cpHdel75-T2A-NLS-EGFP	AAVS1 safe harbor	GGG-Hpep11	CRISPR/Cas9 KI	Supplementary Fig 12d
293T	GGG-HaloTag	CRISPR/Cas9 KI	-	-	Supplementary Fig 12d

Supplementary Table 17. U2OS transient transfection and associated experimental figures.

1st expression cassette (stable expression)	2nd expression cassette (stable expression)	3rd expression cassette (plasmid transient transfection)	Figure	Annotation
EGFP-GSG-cpHaloΔ3	-	H2B-SNAPf-Hpep9	Fig. 2a	
EGFP-GSG-cpHaloΔ3	-	TOMM20-SNAPf-Hpep9	Fig. 2a	
EGFP-GSG-cpHaloΔ3	-	Hpep9-SNAPf-LamB1	Fig. 2a	
EGFP-GSG-cpHaloΔ3	-	H2B-SNAPf-Hpep11	Fig. 2b	
EGFP-GSG-cpHaloΔ3	-	TOMM20-SNAPf-Hpep11	Fig. 2b	
EGFP-GSG-cpHaloΔ3	-	Hpep11-SNAPf-LamB1	Fig. 2b	
-	-	TOMM20-SNAPf-Hpep11	Supplementary Fig 10a	
-	-	EGFP-GSG-cpHaloΔ3	Supplementary Fig 10a	
-	-	Hpep11-SNAPf-LamB1	Supplementary Fig 10b	
EGFP-GSG-cpHaloΔ3	-	Hpep11-SNAPf-LamB1	Supplementary Fig 10b	
cpHaloΔ3-T2A-NLS-EGFP	-	Hpep11-SNAPf-LamB1	Supplementary Fig 10b	
cpHaloΔ3-T2A-NLS-EGFP	TOMM20-SNAPf-Hpep11	H2B-SNAPf-Hpep8	Fig. 5d	
cpHaloΔ3-T2A-NLS-EGFP	TOMM20-SNAPf-Hpep11	H2B-SNAPf-Hpep9	Fig. 5d	
cpHaloΔ3-T2A-NLS-EGFP	TOMM20-SNAPf-Hpep11	H2B-SNAPf-Hpep10	Fig. 5d	
-	-	cpHaloΔ3-T2A-EGFP-P30-SNAPf-NLS3x	Supplementary Fig 11b	Control
-	-	cpHaloΔ3-T2A-EGFP-Hpep11[loopC_gsgx2]-P30-SNAPf-NLS3x	Supplementary Fig 11b	LoopC
-	-	EGFP-cpHaloTag[Hpep11+cpHaloΔ3][loopC_gsgx2]-P30-SNAPf-NLS3x	Supplementary Fig 11b	LoopC
-	-	EGFP-HaloTag7[loopC_gsgx2]-P30-SNAPf-NLS3x	Supplementary Fig 11b	LoopC
-	-	cpHaloΔ3-T2A-EGFP-gsg-Hpep11-P30-SNAPf-NLS3x	Supplementary Fig 11b	C-terminus
-	-	EGFP-gsg-cpHaloTag[Hpep11+cpHaloΔ3]-P30-SNAPf-NLS3x	Supplementary Fig 11b	C-terminus
-	-	EGFP-gsg-HaloTag7-P30-SNAPf-NLS3x	Supplementary Fig 11b	C-terminus
-	-	cpHaloΔ3-T2A-gsg-Hpep11-gsg-EGFP-P30-SNAPf-NLS3x	Supplementary Fig 11b	N-terminus
-	-	cpHaloTag[Hpep11+cpHaloΔ3]-gsg-EGFP-P30-SNAPf-NLS3x	Supplementary Fig 11b	N-terminus
-	-	HaloTag7-gsg-EGFP-P30-SNAPf-NLS3x	Supplementary Fig 11b	N-terminus

All the above-listed mammalian expression plasmids were constructed using the pcDNA5/FRT/TO vector.

Supplementary Table 18. Confocal image acquisition parameters.

Figure	Hpep tagging target/construct (s)	Ligands for labeling (s)	Objectives	Excitation [nm] (laser power/ch.)	Emission [nm]	Pixel dwell time [µs]	Pinhole [Airy units, mAU]	Pixel size [nm]	Size [pixels]	Comments
2a	H2B-STf-Hpep9	CP-TMR, CA-SiR	40x/1.10 water	485 (0.35% EGFP), 550 (0.3% TMR), 640 (0.1% SiR)	495-530, 560-600, 650-720	1.75	999.46	90	928x928	2 line-average
2a	TOM20-STf-Hpep9	CP-TMR, CA-SiR	40x/1.10 water	485 (1.5% EGFP), 550 (1% TMR), 640 (0.5% SiR)	495-530, 560-600, 650-720	1.3875	999.46	90	1168x1168	2 line-average
2a	TOM20-STf-Hpep9 (zoom-in)	CP-TMR, CA-SiR	40x/1.10 water	485 (3% EGFP), 550 (1% TMR), 640 (0.5% SiR)	495-530, 560-600, 650-720	14.5	999.46	87	112x112	2 line-average
2a	Hpep9-STf-LamB1	CP-TMR, CA-SiR	40x/1.10 water	485 (0.3% EGFP), 555 (0.2% TMR), 640 (2% SiR)	495-530, 565-620, 650-720	0.5	999.46	90	3248x3248	2 line-average
2b	TOM20-STf-Hpep11	CP-TMR, CA-SiR	40x/1.10 water	485 (3% EGFP), 555 (1% TMR), 640 (1% SiR)	495-530, 565-620, 650-720	0.7875	999.46	41	2048x2048	2 line-average
2b	H2B-STf-Hpep11	CP-TMR, CA-SiR	40x/1.10 water	485 (10% EGFP), 555 (1% TMR), 640 (1% SiR)	495-530, 565-620, 650-720	0.7875	999.46	57	2048x2048	2 line-average
2b	Hpep11-STf-LamB1	CP-TMR, CA-SiR	40x/1.10 water	485 (4.5% EGFP), 555 (2% TMR), 640 (15% SiR)	495-530, 565-620, 650-720	0.7875	999.46	57	2048x2048	2 line-average
2c	H2B-STf-Hpep11	cpHaloΔ3, CA-SiR, CP-TMR	40x/1.10 water	555 (1% TMR) 645 (2% SiR)	565-600 655-720	0.85	999.46	102	1896x1896	2 line-average

Figure	Hpep tagging target/construct (s)	Ligands for labeling (s)	Objectives	Excitation [nm] (laser power/ch.)	Emission [nm]	Pixel dwell time [µs]	Pinhole [Airy units, mAU]	Pixel size [nm]	Size [pixels]	Comments
2c	TOM20-STf-Hpep11	cpHaloΔ3, CA-SiR, CP-TMR	40x/1.10 water	555 (6% TMR)<br>645 (25% SiR)	565-600<br>655-720	1.575	999.46	142	1024x1024	2 line-average
2c	U2OS blank cells	cpHaloΔ3, CA-SiR, CP-TMR	40x/1.10 water	555 (6% TMR)<br>645 (25% SiR)	565-600<br>655-720	0.85	999.46	102	1896x1896	2 line-average
2d	TOM20-STf-Hpep11	CA-MaP555,	40x/1.10 water	555 (100%)	565-620	1.575	999.46	142	1024x1024	2 line-average
2d	TOM20-STf-Hpep11	CA-TMR	40x/1.10 water	555 (21.25%)	565-620	1.575	999.46	142	1024x1024	2 line-average
2d	TOM20-STf-Hpep11	CA-CPY	40x/1.10 water	610 (7.27%)	620-700	3.8375	999.46	142	1024x1024	2 line-average
3b	Hpep11-Sec61B	CA-CPY	40x/1.10 water	485 (1% EGFP),<br>615 (3.5% CPY)	495-532,<br>625-700	1.4625	999.46	90	1104x1104	2 line-average
3b	Hpep11-VIM	CA-CPY	40x/1.10 water	485 (0.3% EGFP),<br>615 (2% CPY)	495-532,<br>625-700	1.45	999.46	89	1120x1120	2 line-average
3b	Hpep11-CLTA	CA-CPY	40x/1.10 water	485 (0.6% EGFP),<br>615 (1.5% CPY)	495-532,<br>625-700	1.4875	999.46	89	1088x1088	2 line-average
3b	Hpep11-LMNA	CA-CPY	40x/1.10 water	485 (1% EGFP),<br>615 (1.8% CPY)	495-532,<br>625-700	1.2375	999.46	89	1304x1304	2 line-average
3b	HIST2H2BE-Hpep11	CA-CPY	40x/1.10 water	485 (0.7% EGFP),<br>615 (0.3% CPY)	495-532,<br>625-700	0.9875	999.46	89	1640x1640	2 line-average
3b	TOMM20-Hpep11	CA-CPY	40x/1.10 water	485 (2% EGFP),<br>615 (2% CPY)	495-532,<br>625-700	1.2125	999.46	89	1328x1328	2 line-average
Supplementary Fig 12a	Hpep11-LMNA	CA-SiR	40x/1.10 water	485 (1% EGFP),<br>640 (1.5% SiR)	495-532,<br>650-720	1.7	999.46	89	952x952	2 line-average

Figure	Hpep tagging target/construct (s)	Ligands for labeling (s)	Objectives	Excitation [nm] (laser power/ch.)	Emission [nm]	Pixel dwell time [µs]	Pinhole [Airy units, mAU]	Pixel size [nm]	Size [pixels]	Comments
Supplementary Fig 12a	Hpep11-CLTA	CA-SiR	40x/1.10 water	485 (0.3% EGFP), 640 (2% SiR)	495-532, 650-720	1.3	999.46	89	1248x1248	2 line-average
Supplementary Fig 12a	Hpep11-Sec61B	CA-SiR	40x/1.10 water	485 (0.6% EGFP), 640 (3% SiR)	495-532, 650-720	1.2375	999.46	89	1304x1304	2 line-average
Supplementary Fig 12a	HIST2H2BE-Hpep11	CA-SiR	40x/1.10 water	485 (1% EGFP), 640 (0.3% SiR)	495-532, 650-720	1.2125	999.46	89	1336x1336	2 line-average
Supplementary Fig 12a	TOMM20-Hpep11	CA-SiR	40x/1.10 water	485 (0.6% EGFP), 640 (3% SiR)	495-532, 650-720	1.3	999.46	90	1248x1248	2 line-average
3d	TOMM20-Hpep9/Hpep11/HT7	CA-SiR	40x/1.10 water	485 (1.8% EGFP), 640 (10% SiR)	495-530, 650-720	1.4375	999.46	89	1128x1128	
Supplementary Fig 12c	HaloTag-NOP10	CA-SiR	40x/1.10 water	488 (2% EGFP), 640 (3% SiR)	498-520, 650-720	1	999.46	90	1616x1616	
Supplementary Fig 12c	Hpep11-NOP10	CA-SiR	40x/1.10 water	488 (0.5% EGFP), 640 (1% SiR)	498-520, 650-720	1	999.46	90	1616x1616	
3e and Supplementary Fig 12d	HIST2H2BE-Hpep11	CA-TMR, CA-MaP555, CA-JFx608, CA-CPY, CA-MaP618, CA-JF635, CA-SiR, CA-JF646, CA- JF669	40x/1.10 water	488 (1% EGFP), 555 (1% TMR or MaP555), 608 (0.1% JFx608), 610 (0.1% CPY), 618 (2% MaP618), 635 (2% JF635), 640 (0.2% SiR), 646 (0.2% JF646), 669 (0.6% JF669)	498-530, 565-620, 618-720, 620-720, 628-720, 645-720, 650-720, 656-720, 679-720	0.7875	999.46	142	2048x2048	

Figure	Hpep tagging target/construct (s)	Ligands for labeling (s)	Objectives	Excitation [nm] (laser power/ch.)	Emission [nm]	Pixel dwell time [µs]	Pinhole [Airy units, mAU]	Pixel size [nm]	Size [pixels]	Comments
3e and Supplementary Fig 12e	Hpep11-LMNA	CA-TMR,<br>CA-MaP555,<br>CA-JFx608,<br>CA-CPY,<br>CA-MaP618,<br>CA-JF635,<br>CA-SiR,<br>CA-JF646,<br>CA- JF669	40x/1.10 water	488 (1% EGFP),<br>555 (20% TMR or 50% MaP555),<br>608 (0.8% JFx608),<br>610 (0.5% CPY),<br>618 (80% MaP618),<br>635 (80% JF635),<br>640 (8% SiR),<br>646 (15% JF646),<br>669 (50% JF669)	498-530,<br>565-620,<br>618-720,<br>620-720,<br>628-720,<br>645-720,<br>650-720,<br>656-720,<br>679-720	0.7875	999.46	142	2048x2048	
5c	TOMM20-Hpep11	CA-SiR	40x/1.10 water	640 (60% SiR)	650-720	5.125	999.37	118	768x768	CRISPR cells<br>80 MHz, SP8
5c	Hpep11-STf-LamB1	CA-SiR	40x/1.10 water	640 (20% SiR)	650-720	4.2	999.37	118	936x936	80 MHz, SP8
5d	H2B-STf-Hpep10	CA-SiR	40x/1.10 water	640 (3% SiR)	650-720	3.4625	999.37	118	616x616	80 MHz, SP8
5d	H2B-STf-Hpep8	CA-SiR	40x/1.10 water	640 (3% SiR)	650-720	3.425	999.37	118	624x624	80 MHz, SP8
5d	H2B-STf-Hpep9	CA-SiR	40x/1.10 water	640 (1% SiR)	650-720	3.6125	999.37	117	592x592	80 MHz, SP8
Supplementary Fig10a		CP-TMR,<br>CA-SiR	40x/1.10 water	485 (3%),<br>555 (2% TMR),<br>640 (0.2% SiR)	495-530,<br>565-620,<br>650-720	0.7875	999.46	71	2048x2048	
Supplementary Fig10b		CP-TMR,<br>CA-SiR	40x/1.10 water	485 (1.5%),<br>555 (1% TMR),<br>640 (4.5% SiR)	495-530,<br>565-620,<br>650-720	1.5375	999.46	92	1056x1056	

Figure	Hpep tagging target/construct (s)	Ligands for labeling (s)	Objectives	Excitation [nm] (laser power/ch.)	Emission [nm]	Pixel dwell time [µs]	Pinhole [Airy units, mAU]	Pixel size [nm]	Size [pixels]	Comments
Supplementary Fig10c		CP-TMR, CA-CPY	40x/1.10 water	485 (10% EGFP), 550 (1.6% TMR), 600 (1.5% SiR)	495-530, 565-595, 610-700	1.575	999.46	284	1024x1024	Live-cell
Supplementary Fig10c		CP-TMR, CA-CPY	40x/1.10 water	485 (1.5% EGFP), 550 (3.5% TMR), 600 (0.7% SiR)	495-530, 565-595, 610-700	1.575	999.46	142	1024x1024	Fixed cell
Supplementary Fig10d	H2B-STf-Hpep11	cpHalo∆3, CA-CPY, CP-TMR	40x/1.10 water	555 (0.5% TMR), 615 (0.5% CPY)	565-600, 625-720	1.575	999.46	189	1024x1024	
Supplementary Fig10d	TOM20-STf-Hpep11	cpHalo∆3, CA-CPY, CP-TMR	40x/1.10 water	555 (6% TMR), 615 (15% CPY)	565-600, 625-720	1.575	999.46	189	1024x1024	
Supplementary Fig10d	U2OS blank cells	cpHalo∆3, CA-CPY, CP-TMR	40x/1.10 water	555 (6% TMR), 615 (25% CPY)	565-600, 625-720	0.85	999.46	102	1896x1896	
Supplementary Fig11c		CP-TMR, CA-SiR	40x/1.10 water	485 (1.5% EGFP), 550 (0.3% TMR),	495-530, 560-620,	0.7875	999.46	142	2048x2048	

All the above-mentioned image acquisitions were performed on Stellaris 5 confocal microscope unless otherwise stated.

Supplementary Table 19. Confocal microscope laser powers.

Excitation wavelength [nm]	Laser power at 100% output [μW]	Objective	System laser output setting
485	80	40x/1.10 water	85%
488	90	40x/1.10 water	85%
550	220	40x/1.10 water	85%
555	180	40x/1.10 water	85%
608	315	40x/1.10 water	85%
610	320	40x/1.10 water	85%
615	330	40x/1.10 water	85%
618	340	40x/1.10 water	85%
635	370	40x/1.10 water	85%
640	390	40x/1.10 water	85%
645	400	40x/1.10 water	85%
646	403	40x/1.10 water	85%
669	420	40x/1.10 water	85%

Laser power was measured using an optical power meter (Thorlabs) for all wavelengths used in this study with a 40× water-immersion objective. Measurements were taken at 100% laser output, which corresponds to 85% of the maximum laser power.

Supplementary Table 20. STED image acquisition parameters.

Figure	Target	Cell-lines	Imaging	Ex (%)	STED (%)	Pixel dwell time [µs]	Pixel size [nm]	Size [µm]	Emission [nm]	Comments
4a (upper)	TOM20-STf-Hpep11 + EGFP-GSG-cpHaloΔ3	KI	CLSM<br>STED	10<br>13	-<br>10	15	30	10 x 10	650-757	3 line accu.
4a (lower)	TOM20-GGG-Hpep11 + cpHaloΔ3-T2A-NLS-EGFP	FTR	CLSM<br>STED	10<br>13	-<br>10	15	30	10 x 10	650-757	3 line accu.
Supplementary Fig. 5	TOM20-STf-Hpep11 + EGFP-GSG-cpHaloΔ3 or TOM20-HT7	KI	CLSM<br>STED	10<br>13	-<br>10	15	30	10 x 10	650-757	3 line accu.
4b (upper)	TOM20-HT7	KI	CLSM<br>STED	14<br>18	-<br>10	15	30	10 x 10	650-757	3 line accu.
4b (lower)	TOM20-GGG-Hpep11 + cpHaloΔ3-T2A-NLS-EGFP	KI	CLSM<br>STED	14<br>18	-<br>10	15	30	10 x 10	650-757	3 line accu.
4c	Hpep11-GGG-CLTA + cpHaloΔ3-T2A-NLS-EGFP	KI	CLSM ov<br>CLSM z<br>STED	5<br>5<br>15	-<br>-<br>10	15	80<br>50<br>30	50 x 50<br>10 x 10<br>10 x 10	650-757	2 line accu.
4d (left)	Hpep11-GGG-TUBB4B + cpHaloΔ3-T2A-NLS-EGFP	KI	CLSM ov<br>CLSM z<br>STED	2<br>3<br>5	-<br>-<br>20	10	80<br>50<br>30	50 x 50<br>10 x 10<br>10 x 10	650-757	3 line accu.
4d (right)	HaloTag7-GGG-TUBB4B	KI	CLSM ov<br>CLSM z<br>STED	2<br>3<br>5	-<br>-<br>20	10	80<br>50<br>30	50 x 50<br>10 x 10<br>10 x 10	650-757	3 line accu.

Excitation line: 640 nm; STED line: 775 nm. KI – Knock-in, FTR – overexpression via Flp-In T-REx system. ov – overview, z – zoom.

Supplementary Table 21. STED microscope laser powers.

	Laser input [%]	100	50	40	20	10	5	1
Laser output	640 nm excitation [µW]	405.3 ± 4.7	-	-	-	37.9 ± 0.1	18.5 ± 0.2	3.0 ± 0.1
	775 nm STED [mW]	831.7± 54.8	416.0 ± 26.5	330.3 ± 21.5	165.0 ± 11.4	81.4 ± 5.7	39.5 ± 2.7	7.9 ± 0.5

Average data and standard deviation from 3 measurements during the time of experiments. The laser powers were measured at the back focal aperture without the objective lense.

Supplementary Table 22. Summary of binding and fluorescence properties of Hpep variants in complex with cpHaloΔ3.

Hpep	a. Labeling kinetics (FP)	b. Binding (FP)	c. Binding (FP)	d. Binding kinetics (BLI)	e. Binding kinetics (BLI)	f. Hpep-induced fluo. turn-on of	
	EC_{50} (with cpHaloΔ3)	K_d (with cpHaloΔ3)	K_d (with cpHaloΔ3-SiR)	K_d (with cpHaloΔ3-SiR)	K_d (with cpHaloΔ3-TMR)	cpHaloΔ3-SiR	cpHaloΔ3-CPY
**Hpep7**	62 nM	*n.d.*	*n.d.*	*n.d.*	*n.d.*	1.9x	6.2x
**Hpep8**	14 nM	*n.d.*	*n.d.*	*n.d.*	*n.d.*	1.7x	4.5x
**Hpep9**	1.7 nM	6.5 nM	3.3 nM	18.8 nM	4.86 nM	1.5x	3.0x
**Hpep10**	2.1 nM	*n.d.*	*n.d.*	*n.d.*	*n.d.*	1.5x	2.4x
**Hpep11**	25 nM	14 nM	1.3 nM	4.27 nM	10.6 nM	1.9x	6.1x
**Hpep format**	Untagged Hpep	TMR-Hpep	TMR-Hpep	Biotin-Hpep	Biotin-Hpep	Untagged Hpep	

Abbreviations: K_d: dissociation constant; EC_{50}: Hpep concentration required to achieve half-maximum labeling speed of cpHaloΔ; fluo.: fluorescence *n.d.: not determined*

(a.) The values are compiled from Figure 1 and Supplementary Fig 3.
(b-e.) The data are compiled from Supplementary Fig 7.
(f.) Fold-change values are compiled from Figure 1 and Supplementary Fig 6.

In general, the binding properties of Hpep with cpHaloΔ3 show consistent trends across different assays. Specifically, we characterized Hpep9 and Hpep11 in complex with either unlabeled or SiR-labeled cpHaloΔ3 using two independent assays. The results were in good agreement: Hpep9 showed stronger binding than Hpep11 to unlabeled-cpHaloΔ3, but lower affinity to SiR-labeled cpHaloΔ3. The variations in binding parameters across assays likely reflect differences in Hpep format and measurement conditions. For FP assays, Hpep was fluorophore-labeled (TMR), which may introduce additional interactions with cpHaloΔ3. For BLI assays, Hpep was immobilized via biotin, potentially limiting accessibility. In contrast, labeling kinetics performed in solution with unmodified components represents the ideal measurement, but only yields EC_{50} values, or in other words, labeling affinities.

Protein sequences

Expression in *E.coli*

>cpHaloΔ

MHHHHHHHHHHENLYFQGDVGRKLIIDQNVFIEGTLPMGVVRPLTEVEMDHYREPFLNPVDREPLWRFPNELPIAGEPANIV
ALVEEYMDWLHQSPVPKLLFWGTPGVLIPPAEAARLAKSLPNCKAVDIGPGLNLLQEDNPDLIGSEIARWLSTLEIGGTGGSGGT
GGSGGSIGTGFPFDPHYVEVLGERMHYVDVGPRDGTPVLFLHGNPTSSYVWRNIIPHVAPTHRCIAPDLIGMGKSDKPDLGYFFD
DHVRFMDAFIEALGLEEVVLVIHDWGSALGFHWAKRNPERVKGIAFMEFIRPIPTWDEW*

His-tag – TEVp site – cpHaloΔ

>cpHaloΔ2

MHHHHHHHHHHENLYFQGDVGRKLIIDQNVFIEGTLPMGVVRPLTEEEMDHYREPFLNPKDREPLWRFPNELPIAGEPANIV
ALVEEYMDWLHQSPVPKLLFWGTPGVLIPPAEAARLAKSLPNCKAVDIGPGLNLLQEDNPDLIGSEIARWLSTLEIKSKYDRDQI
LKIIAELEKKTGGSIGTGFPFDPHYVEVLGSRMHYVDVGPRDGTPVLFLHGNPTSSYVWRNIIPHVAPTHRCIAPDLIGMGKSDKP
DLGYFFDDHVRFMDAFIEALGLEEVVLVIHDWGSALGFHWAKRHPERVKGIAFMEFIRPIPTWDEW*

His-tag – TEVp site – cpHaloΔ2

>cpHaloΔ3

MHHHHHHHHHHENLYFQGEKKGDVGRKLIIDQNVFIEGTLPMGVVRPLTEEEMDHYREPFLNPKDREPLWRFPNELPIAGEP
ANIVALVEEYMDWLHQSPVPKLLFWGTPGVLIPPAEAARLAKSLPNCKAVDIGPGLNLLQEDNPDLIGSEIARWLSTLEIKSKYD
RDQILKIIAELEKKTGGSIGTGFPFDPHYVEVLGSRMHYVDVGPRDGTPVLFLHGNPTSSYVWRNIIPHVAPTHRCIAPDLIGMGK
SDKPDLGYFFDDHVRFMDAFIEALGLEEVVLVIHDWGSALGFHWAKRHPERVKGIAFMEFIRPIPTWDEWGDVE*

His-tag – TEVp site – cpHaloΔ3

Expression on yeast surface

>pJYDNg-cpHaloΔ_N-term extension

MRFPSIFTAVVFAASSALAAPANGTMVGSGXXXXDVGRKLIIDQNVFIEGTLPMGVVRPLTEVEMDHYREPFLNPVDREPLWRF
PNELPIAGEPANIVALVEEYMDWLHQSPVPKLLFWGTPGVLIPPAEAARLAKSLPNCKAVDIGPGLNLLQEDNPDLIGSEIARWL
STLEIGGTGGSGGTGGSGGSIGTGFPFDPHYVEVLGERMHYVDVGPRDGTPVLFLHGNPTSSYVWRNIIPHVAPTHRCIAPDLIG
MGKSDKPDLGYFFDDHVRFMDAFIEALGLEEVVLVIHDWGSALGFHWAKRNPERVKGIAFMEFIRPIPTWDEWAAAFSQKLDI
NLLDNVVNSSYHGEGVSGGSAQELTTICEQIPSPTLESTPYSLSTTTILANGKAMQGVFEYYKSVTFVSNCGSHPSTTSKGSPINTQ
YVFKDNSSTIEGRYPYDVPDYALQASGGGGSGGGGSGGGGSASHEQKLISEEDLMLEKFVGTWKIESSENFGEYLKAIGAPKELAD
AGDATTPVLYISQKDGDKMTVKIENGPPTFLDTQVSFKLGEEFDEFPSDRRKGVKSVVNLSGEKLVYVQKWDGKETTYVREIKD
GKLVVTLTMGDVVAVRSYRRASE**

Leader sequence appS4 – cpHaloΔ_Lib – Aga2p – Factor Xa site – HA-tag – Myc-tag – Linker – eUnaG2

>pJYDNg-cpHaloΔ_C-term extension

MRFPSIFTAVVFAASSALAAPANGTMVDVGRKLIIDQNVFIEGTLPMGVVRPLTEVEMDHYREPFLNPVDREPLWRFPNELPIA
GEPANIVALVEEYMDWLHQSPVPKLLFWGTPGVLIPPAEAARLAKSLPNCKAVDIGPGLNLLQEDNPDLIGSEIARWLSTLEIGG
TGGSGGTGGSGGSIGTGFPFDPHYVEVLGERMHYVDVGPRDGTPVLFLHGNPTSSYVWRNIIPHVAPTHRCIAPDLIGMGKSDKP
DLGYFFDDHVRFMDAFIEALGLEEVVLVIHDWGSALGFHWAKRNPERVKGIAFMEFIRPIPTWDEWXXXXAAAFSQKLDINLL
DNVVNSSYHGEGVSGGSAQELTTICEQIPSPTLESTPYSLSTTTILANGKAMQGVFEYYKSVTFVSNCGSHPSTTSKGSPINTQYVF
KDNSSTIEGRYPYDVPDYALQASGGGGSGGGGSGGGGSASHEQKLISEEDLMLEKFVGTWKIESSENFGEYLKAIGAPKELADAG
DATTPVLYISQKDGDKMTVKIENGPPTFLDTQVSFKLGEEFDEFPSDRRKGVKSVVNLSGEKLVYVQKWDGKETTYVREIKDGK
LVVTLTMGDVVAVRSYRRASE**

Leader sequence appS4 – cpHaloΔ_Lib – Aga2p – Factor Xa site – HA-tag – Myc-tag – Linker – eUnaG2

Expression in mammalian cells

> cpHaloΔ

DVGRKLIIDQNVFIEGTLPMGVVRPLTEVEMDHYREPFLNPVDREPLWRFPNELPIAGEPANIVALVEEYMDWLHQSPVPKLLF
WGTPGVLIPPAEAARLAKSLPNCKAVDIGPGLNLLQEDNPDLIGSEIARWLSTLEIGGTGGSGGTGGSGGSIGTGFPFDPHYVEVL
GERMHYVDVGPRDGTPVLFLHGNPTSSYVWRNIIPHVAPTHRCIAPDLIGMGKSDKPDLGYFFDDHVRFMDAFIEALGLEEVVL
VIHDWGSALGFHWAKRNPERVKGIAFMEFIRPIPTWDEW

> cpHaloΔ2

DVGRKLIIDQNVFIEGTLPMGVVRPLTEEEMDHYREPFLNPKDREPLWRFPNELPIAGEPANIVALVEEYMDWLHQSPVPKLLF
WGTPGVLIPPAEAARLAKSLPNCKAVDIGPGLNLLQEDNPDLIGSEIARWLSTLEIKSKYDRDQILKIIAELEKKTGGSIGTGFPFD
PHYVEVLGSRMHYVDVGPRDGTPVLFLHGNPTSSYVWRNIIPHVAPTHRCIAPDLIGMGKSDKPDLGYFFDDHVRFMDAFIEAL
GLEEVVLVIHDWGSALGFHWAKRHPERVKGIAFMEFIRPIPTWDEW

> cpHaloΔ3

EKKGDVGRKLIIDQNVFIEGTLPMGVVRPLTEEEMDHYREPFLNPKDREPLWRFPNELPIAGEPANIVALVEEYMDWLHQSPVP
KLLFWGTPGVLIPPAEAARLAKSLPNCKAVDIGPGLNLLQEDNPDLIGSEIARWLSTLEIKSKYDRDQILKIIAELEKKTGGSIGTG
FPFDPHYVEVLGSRMHYVDVGPRDGTPVLFLHGNPTSSYVWRNIIPHVAPTHRCIAPDLIGMGKSDKPDLGYFFDDHVRFMDA
FIEALGLEEVVLVIHDWGSALGFHWAKRHPERVKGIAFMEFIRPIPTWDEWGDVE
N/C-terminal extensions – cpHaloΔ2

> HaloTag

GSEIGTGFPFDPHYVEVLGERMHYVDVGPRDGTPVLFLHGNPTSSYVWRNIIPHVAPTHRCIAPDLIGMGKSDKPDLGYFFDDH
VRFMDAFIEALGLEEVVLVIHDWGSALGFHWAKRNPERVKGIAFMEFIRPIPTWDEWPEFARETFQAFRTTDVGRKLIIDQNVF
IEGTLPMGVVRPLTEVEMDHYREPFLNPVDREPLWRFPNELPIAGEPANIVALVEEYMDWLHQSPVPKLLFWGTPGVLIPPAE
AARLAKSLPNCKAVDIGPGLNLLQEDNPDLIGSEIARWLSTLEISG*

>pcDNA5/FRT/TO-cpHaloΔ-T2A-NLS-EGFP

M[cpHaloΔ]GSGATNFSLLKQAGDVEENPGPSRMAPKKKRKVMVSKGEELFTGVVPILVELDGDVNGHKFSVSGEGEGDATYGK
LTLKFICTTGKLPVPWPTLVTTLTYGVQCFSRYPDHMKQHDFFKSAMPEGYVQERTIFFKDDGNYKTRAEVKFEGDTLVNRIEL
KGIDFKEDGNILGHKLEYNYNSHNVYIMADKQKNGIKVNFKIRHNIEDGSVQLADHYQQNTPIGDGPVLLPDNHYLSTQSALSK
DPNEKRDHMVLLEFVTAAGITLGMDELYK**
cpHaloΔ – T2A (2A self-cleaving sequence) – NLS (nuclear localization signal or sequence) – Linker – EGFP

>pcDNA5/FRT/TO-EGFP-cpHaloΔ

MVSKGEELFTGVVPILVELDGDVNGHKFSVSGEGEGDATYGKLTLKFICTTGKLPVPWPTLVTTLTYGVQCFSRYPDHMKQHDF
FKSAMPEGYVQERTIFFKDDGNYKTRAEVKFEGDTLVNRIELKGIDFKEDGNILGHKLEYNYNSHNVYIMADKQKNGIKVNFKI
RHNIEDGSVQLADHYQQNTPIGDGPVLLPDNHYLSTQSALSKDPNEKRDHMVLLEFVTAAGITLGMDELYKGSGGTGGSG[cpH
aloΔ]*
EGFP – Linker – cpHaloΔ

>pcDNA5/FRT/TO-H2B-SNAPf-Hpep

MPEPAKSAPAPKKGSKKAVTKAQKKGGKKRKRSRKESYSIYVYKVLKQVHPDTGISSKAMGIMNSFVNDIFERIAGEASRLAHY
NKRSTITSREIQTAVRLLLPGELAKHAVSEGTKAITKYTSAKGGDKDCEMKRTTLDSPLGKLELSGCEQGLHRIIFLGKGTSAADA
VEVPAPAAVLGGPEPLMQATAWLNAYFHQPEAIEEFPVPALHHPVFQQESFTRQVLWKLLKVVKFGEVISYSHLAALAGNPAA
TAAVKTALSGNPVPILIPCHRVVQGDLDVGGYEGGLAVKEWLLAHEGHRLGKPGLGSG[Hpep]*
H2B – Linker – SNAPf – Hpep

>pcDNA5/FRT/TO-LamB1-SNAPf-Hpep

MATATPVPPRMGSRAGGPTTPLSPTRLSRLQEKEELRELNDRLAVYIDKVRSLETENSALQLQVTEREEVRGRELTGLKALYET
ELADARRALDDTARERAKLQIELGKCKAEHDQLLLNYAKKESDLNGAQIKLREYEAALNSKDAALATALGDKKSLEGDLEDLKD
QIAQLEASLAAAKKQLADETLLKVDLENRCQSLTEDLEFRKSMYEEEINETRRKHETRLVEVDSGRQIEYEYKLAQALHEMREQ
HDAQVRLYKEELEQTYHAKLENARLSSEMNTSTVNSAREELMESRMRIESLSSQLSNLQKESRACLERIQELEDLLAKEKDNSR
RMLTDKEREMAEIRDQMQQQLNDYEQLLDVKLALDMEISAYRKLLEGEEERLKLSPSPSSRVTVSRASSSRSVRTTRGKRKRVD
VEESEASSSVSISHSASATGNVCIEEIDVDGKFIRLKNTSEQDQPMGGWEMIRKIGDTSVSYKYTSRYVLKAGQTVTIWAANAGVT
ASPPTDLIWKNQNSWGTGEDVKVILKNSQGEEVAQRSTVFKTTIPEEEEEEEEAAGVVVEEELFHQQGTPRASNRSCAIMGGDK
DCEMKRTTLDSPLGKLELSGCEQGLHRIIFLGKGTSAADAVEVPAPAAVLGGPEPLMQATAWLNAYFHQPEAIEEFPVPALHHP
VFQQESFTRQVLWKLLKVVKFGEVISYSHLAALAGNPAATAAVKTALSGNPVPILIPCHRVVQGDLDVGGYEGGLAVKEWLLAH
EGHRLGKPGLGSG[Hpep]*
LamB1 – Linker – SNAPf – Hpep

>pcDNA5/FRT/TO-TOMM20-SNAPf-Hpep

MVGRNSAIAAGVCGALFIGYCIYFDRKRRSDPNFKNRLRERRKKQKLAKERAGLSKLPDLKDAEAVQKFFLEEIQLGEELLAQGE
YEKGVDHLTNAIAVCGQPQQLLQVLQQTLPPPVFQMLLTKLPTISQRIVSAQSLAEDDVEGGSGDPPVGGDKDCEMKRTTLDSP
LGKLELSGCEQGLHRIIFLGKGTSAADAVEVPAPAAVLGGPEPLMQATAWLNAYFHQPEAIEEFPVPALHHPVFQQESFTRQVL
WKLLKVVKFGEVISYSHLAALAGNPAATAAVKTALSGNPVPILIPCHRVVQGDLDVGGYEGGLAVKEWLLAHEGHRLGKPGLGS
G[Hpep]*
TOMM20 – Linker – SNAPf – Hpep

>pcDNA5/FRT/TO-cpHaloΔ3-T2A-EGFP-P30-SNAPf-NLS3x (cpHaloΔ3 only)

M[cpHaloΔ3]GSGATNFSLLKQAGDVEENPGPMVSKGEELFTGVVPILVELDGDVNGHKFSVSGEGEG
DATYGKLTLKFICTTGKLPVPWPTLVTTLTYGVQCFSRYPDHMKQHDFFKSAMPEGYVQERTIFFKDDGNYKTRAEVKFEGDT
LVNRIELKGIDFKEDGNILGHKLEYNYNSHNVYIMADKQKNGIKVNFKIRHNIEDGSVQLADHYQQNTPIGDGPVLLPDNHYLST
QSALSKDPNEKRDHMVLLEFVTAAGITLGMDELYKSGRPPPPPPPPPPPPPPPPPPPPPPPPPPPPPPGGRSRSLEMDKDCEMK
RTTLDSPLGKLELSGCEQGLHRIIFLGKGTSAADAVEVPAPAAVLGGPEPLMQATAWLNAYFHQPEAIEEFPVPALHHPVFQQES
FTRQVLWKLLKVVKFGEVISYSHLAALAGNPAATAAVKTALSGNPVPILIPCHRVVQGDLDVGGYEGGLAVKEWLLAHEGHRLG
KPGLGGAPDPKKKRKVDPKKKRKVDPKKKRKEL

T2A – Linker – EGFP – SNAPf – NLS 3x

>pcDNA5/FRT/TO-cpHaloΔ3-T2A-gsg-Hpep11-gsg-EGFP-P30-SNAPf-NLS3x (N-terminal tagging)

M[cpHaloΔ3]GSGATNFSLLKQAGDVEENPGPGSGSKRDWREMFRLFRTGSGMVSKGEELFTGVVPILVELDGDVNGHKFSVSG
EGEGDATYGKLTLKFICTTGKLPVPWPTLVTTLTYGVQCFSRYPDHMKQHDFFKSAMPEGYVQERTIFFKDDGNYKTRAEVKF
EGDTLVNRIELKGIDFKEDGNILGHKLEYNYNSHNVYIMADKQKNGIKVNFKIRHNIEDGSVQLADHYQQNTPIGDGPVLLPDN
HYLSTQSALSKDPNEKRDHMVLLEFVTAAGITLGMDELYKSGRPPPPPPPPPPPPPPPPPPPPPPPPPPPPPPGGRSRSLEMDKD
CEMKRTTLDSPLGKLELSGCEQGLHRIIFLGKGTSAADAVEVPAPAAVLGGPEPLMQATAWLNAYFHQPEAIEEFPVPALHHPV
FQQESFTRQVLWKLLKVVKFGEVISYSHLAALAGNPAATAAVKTALSGNPVPILIPCHRVVQGDLDVGGYEGGLAVKEWLLAHE
GHRLGKPGLGGAPDPKKKRKVDPKKKRKVDPKKKRKEL*

T2A – Linker – EGFP – Hpep11 – SNAPf – NLS 3x

>pcDNA5/FRT/TO-cpHaloΔ3-T2A-EGFP-gsg-Hpep11-P30-SNAPf-NLS3x (C-terminal tagging)

M[cpHaloΔ3]GSGATNFSLLKQAGDVEENPGPMVSKGEELFTGVVPILVELDGDVNGHKFSVSGEGEGDATYGKLTLKFICTTGK
LPVPWPTLVTTLTYGVQCFSRYPDHMKQHDFFKSAMPEGYVQERTIFFKDDGNYKTRAEVKFEGDTLVNRIELKGIDFKEDGNI
LGHKLEYNYNSHNVYIMADKQKNGIKVNFKIRHNIEDGSVQLADHYQQNTPIGDGPVLLPDNHYLSTQSALSKDPNEKRDHMV
LLEFVTAAGITLGMDELYKGSGSKRDWREMFRLFRTSGRPPPPPPPPPPPPPPPPPPPPPPPPPPPPPPGGRSRSLEMDKDCEM
KRTTLDSPLGKLELSGCEQGLHRIIFLGKGTSAADAVEVPAPAAVLGGPEPLMQATAWLNAYFHQPEAIEEFPVPALHHPVFQQ
ESFTRQVLWKLLKVVKFGEVISYSHLAALAGNPAATAAVKTALSGNPVPILIPCHRVVQGDLDVGGYEGGLAVKEWLLAHEGHR
LGKPGLGGAPDPKKKRKVDPKKKRKVDPKKKRKEL*

T2A – Linker – EGFP – Hpep11 – SNAPf – NLS 3x

>pcDNA5/FRT/TO-cpHaloΔ3-T2A-EGFP-Hpep11[loopC_gsgx2]-P30-SNAPf-NLS3x (LoopC insertion)

M[cpHaloΔ3]GSGATNFSLLKQAGDVEENPGPMVSKGEELFTGVVPILVELDGDVNGHKFSVSGEGEGDATYGKLTLKFICTTGK
LPVPWPTLVTTLTYGVQCFSRYPDHMKQHDFFKSAMPEGYVQERTIFFKDDGNYKTRAEVKFEGDTLVNRIELKGIDFKEDGNI
LGHKLEYNYNSHNVYIMADKQKNGIKVNFKIRHNIEDGSGGSGSKRDWREMFRLFRTGSGGTGVQLADHYQQNTPIGDGPVLL
PDNHYLSTQSALSKDPNEKRDHMVLLEFVTAAGITLGMDELYKSGRPPPPPPPPPPPPPPPPPPPPPPPPPPPPPPGGRSRSLEM
DKDCEMKRTTLDSPLGKLELSGCEQGLHRIIFLGKGTSAADAVEVPAPAAVLGGPEPLMQATAWLNAYFHQPEAIEEFPVPALH
HPVFQQESFTRQVLWKLLKVVKFGEVISYSHLAALAGNPAATAAVKTALSGNPVPILIPCHRVVQGDLDVGGYEGGLAVKEWLL
AHEGHRLGKPGLGGAPDPKKKRKVDPKKKRKVDPKKKRKEL*

T2A – Linker – EGFP – Hpep11 – SNAPf – NLS 3x

>pcDNA5/FRT/TO-EGFP-DogTag[loopC_gsgx2]-P30-SNAPf-NLS3x (LoopC insertion)

MVSKGEELFTGVVPILVELDGDVNGHKFSVSGEGEGDATYGKLTLKFICTTGKLPVPWPTLVTTLTYGVQCFSRYPDHMKQHDF
FKSAMPEGYVQERTIFFKDDGNYKTRAEVKFEGDTLVNRIELKGIDFKEDGNILGHKLEYNYNSHNVYIMADKQKNGIKVNFKI
RHNIEDGTGGSGDIPATYEFTDGKHYITNEPIPPKGSGGTGVQLADHYQQNTPIGDGPVLLPDNHYLSTQSALSKDPNEKRDHM
VLLEFVTAAGITLGMDELYKSGRPPPPPPPPPPPPPPPPPPPPPPPPPPPPPPGGRSRSLEMDKDCEMKRTTLDSPLGKLELSGCE
QGLHRIIFLGKGTSAADAVEVPAPAAVLGGPEPLMQATAWLNAYFHQPEAIEEFPVPALHHPVFQQESFTRQVLWKLLKVVKFG
EVISYSHLAALAGNPAATAAVKTALSGNPVPILIPCHRVVQGDLDVGGYEGGLAVKEWLLAHEGHRLGKPGLGGAPDPKKKRKV
DPKKKRKVDPKKKRKEL*

Linker – EGFP – DogTag – SNAPf – NLS 3x

References

1 Wilhelm, J., Nickel, L., Lin, Y.-H., Hiblot, J. & Johnsson, K. Improving Split-HaloTag through Computational Protein Engineering. *bioRxiv*, doi:10.1101/2024.10.13.617931 (2024).

2 Abramson, J. *et al.* Accurate structure prediction of biomolecular interactions with AlphaFold 3. *Nature* **630**, 493-500, doi:10.1038/s41586-024-07487-w (2024).

3 Keeble, A. H. *et al.* DogCatcher allows loop-friendly protein-protein ligation. *Cell Chem Biol* **29**, 339-350 e310, doi:10.1016/j.chembiol.2021.07.005 (2022).

4 Huppertz, M. C. *et al.* Recording physiological history of cells with chemical labeling. *Science* **383**, 890-897, doi:10.1126/science.adg0812 (2024).

5 Uhlen, M. *et al.* Proteomics. Tissue-based map of the human proteome. *Science* **347**, 1260419, doi:10.1126/science.1260419 (2015).

6 Wang, L. *et al.* A general strategy to develop cell permeable and fluorogenic probes for multicolour nanoscopy. *Nat Chem* **12**, 165-+, doi:10.1038/s41557-019-0371-1 (2020).

7 Lin, D. *et al.* Time-tagged ticker tapes for intracellular recordings. *Nat Biotechnol* **41**, 631-639, doi:10.1038/s41587-022-01524-7 (2023).

8 Grimm, J. B. *et al.* A general method to fine-tune fluorophores for live-cell and in vivo imaging. *Nat Methods* **14**, 987-994, doi:10.1038/nmeth.4403 (2017).

9 Mudd, G. *et al.* A general synthetic route to isomerically pure functionalized rhodamine dyes. *Methods Appl Fluoresc* **3**, 045002, doi:10.1088/2050-6120/3/4/045002 (2015).

10 Wang, L. *et al.* A general strategy to develop cell permeable and fluorogenic probes for multicolour nanoscopy. *Nat Chem* **12**, 165-172, doi:10.1038/s41557-019-0371-1 (2020).

11 Grimm, J. B. *et al.* A General Method to Improve Fluorophores Using Deuterated Auxochromes. *JACS Au* **1**, 690-696, doi:10.1021/jacsau.1c00006 (2021).

12 Butkevich, A. N. *et al.* Fluorescent Rhodamines and Fluorogenic Carbopyronines for Super-Resolution STED Microscopy in Living Cells. *Angew Chem Int Ed Engl* **55**, 3290-3294, doi:10.1002/anie.201511018 (2016).

13 Lukinavicius, G. *et al.* A near-infrared fluorophore for live-cell super-resolution microscopy of cellular proteins. *Nat Chem* **5**, 132-139, doi:10.1038/nchem.1546 (2013).

14 Grimm, J. B. *et al.* A general method to improve fluorophores for live-cell and single-molecule microscopy. *Nat Methods* **12**, 244-250, 243 p following 250, doi:10.1038/nmeth.3256 (2015).
