# Supplementary material for: A high-affinity split-HaloTag for live-cell protein labeling
Source: Nat Commun. 2026 Mar 25;17:2865. doi: 10.1038/s41467-026-71032-8 (PMC13022382; doi:10.1038/s41467-026-71032-8)
Supplement: Supplementary file 2 — Reporting Summary [file 41467_2026_71032_MOESM2_ESM.pdf]

nature portfolio

Corresponding author(s): Dr. Julien Hiblot, Prof. Dr. Kai Johnsson

Last updated by author(s): 24.02.2026

Reporting Summary

Nature Portfolio wishes to improve the reproducibility of the work that we publish. This form provides structure for consistency and transparency in reporting. For further information on Nature Portfolio policies, see our Editorial Policies and the Editorial Policy Checklist.

Statistics

For all statistical analyses, confirm that the following items are present in the figure legend, table legend, main text, or Methods section.

n/a	Confirmed	
☐	☒	The exact sample size (*n*) for each experimental group/condition, given as a discrete number and unit of measurement
☐	☒	A statement on whether measurements were taken from distinct samples or whether the same sample was measured repeatedly
☐	☒	The statistical test(s) used AND whether they are one- or two-sided<br>*Only common tests should be described solely by name; describe more complex techniques in the Methods section.*
☒	☐	A description of all covariates tested
☒	☐	A description of any assumptions or corrections, such as tests of normality and adjustment for multiple comparisons
☐	☒	A full description of the statistical parameters including central tendency (e.g. means) or other basic estimates (e.g. regression coefficient) AND variation (e.g. standard deviation) or associated estimates of uncertainty (e.g. confidence intervals)
☐	☒	For null hypothesis testing, the test statistic (e.g. *F*, *t*, *r*) with confidence intervals, effect sizes, degrees of freedom and *P* value noted<br>*Give P values as exact values whenever suitable.*
☒	☐	For Bayesian analysis, information on the choice of priors and Markov chain Monte Carlo settings
☒	☐	For hierarchical and complex designs, identification of the appropriate level for tests and full reporting of outcomes
☒	☐	Estimates of effect sizes (e.g. Cohen's *d*, Pearson's *r*), indicating how they were calculated

Our web collection on statistics for biologists contains articles on many of the points above.

Software and code

Policy information about availability of computer code

Data collection	Plate reader: Tecan Sparkcontrol Method Editor Version 2.2<br>Flow cytometry: BD Fortessa X-20 Cell Analyzer (BD FACSDiva software)<br>FACS: BD FACSMelody Cell Sorter (BD FACSChrous)<br>Thermal stability: Prometheus NT48 nanoscale differential scanning fluorimeter (R.ThermControl v.2.12)<br>Confocal Microscopy: Leica Stellaris 5 (Leica LAS X 4.5.0.25531) and Leica STELLARIS 8 FALCON microscope (Leica LAS X)<br>STED Microscopy: Abberior STED Expert Line 595/775/RESOLFT QUAD scanning microscope<br>Schematic drawings: BioRender (https://www.biorender.com)<br>Bio-layer interferometry: Sartorius Octet R4 system (Sartorius Octet Analysis Studio)<br>MD simulation: Desmond Molecular Dynamics System (Schrödinger, LLC, New York, NY, 2024-4)
Data analysis	GraphPad Prism (version 9.2.0, 10.3.0), ImageJ2 (version 2.3.0), Fiji (version 1.54f), FlowJo (version 10.7.2), R studio (version 4.1.0), Microsoft Excel (version 16.78.3), Geneious (2021.2.2), AlphaFold3, Simulation Interaction Diagram tool in Maestro (14.2), PyMol (version 3.1.3)

For manuscripts utilizing custom algorithms or software that are central to the research but not yet described in published literature, software must be made available to editors and reviewers. We strongly encourage code deposition in a community repository (e.g. GitHub). See the Nature Portfolio guidelines for submitting code & software for further information.

Data

Policy information about availability of data

All manuscripts must include a data availability statement. This statement should provide the following information, where applicable:

- Accession codes, unique identifiers, or web links for publicly available datasets
- A description of any restrictions on data availability
- For clinical datasets or third party data, please ensure that the statement adheres to our policy

All data supporting the main findings of this study are included in the article and supplementary information. Reagents and materials can be obtained from K.J. upon request. The R script used for the labeling kinetics data analysis was adapted from the script available at https://github.com/Jonas-Wilhelm/jwtools/.

Research involving human participants, their data, or biological material

Policy information about studies with human participants or human data. See also policy information about sex, gender (identity/presentation), and sexual orientation and race, ethnicity and racism.

Reporting on sex and gender	N/A
Reporting on race, ethnicity, or other socially relevant groupings	N/A
Population characteristics	N/A
Recruitment	N/A
Ethics oversight	N/A

Note that full information on the approval of the study protocol must also be provided in the manuscript.

Field-specific reporting

Please select the one below that is the best fit for your research. If you are not sure, read the appropriate sections before making your selection.

- [x] Life sciences
- [ ] Behavioural & social sciences
- [ ] Ecological, evolutionary & environmental sciences

For a reference copy of the document with all sections, see nature.com/documents/nr-reporting-summary-flat.pdf

Life sciences study design

All studies must disclose on these points even when the disclosure is negative.

Sample size	Sample sizes were determined based on experience from previous studies or continued until a clear trend emerged.
Data exclusions	No data was excluded.
Replication	All the replications were successful and the exact number of replicates are stated in the corresponding figure legends or in the Method section. Imaging experiments were conducted on more than one independent sample preparations and distinct field of views.
Randomization	Randomization was not relevant for this study, as no clinical trials are involved.
Blinding	Blinding was not relevant for this study, as no clinical trials are involved.

Reporting for specific materials, systems and methods

We require information from authors about some types of materials, experimental systems and methods used in many studies. Here, indicate whether each material, system or method listed is relevant to your study. If you are not sure if a list item applies to your research, read the appropriate section before selecting a response.

Materials & experimental systems

n/a	Involved in the study
x	Antibodies
	x Eukaryotic cell lines
x	Palaeontology and archaeology
x	Animals and other organisms
x	Clinical data
x	Dual use research of concern
x	Plants

Methods

n/a	Involved in the study
x	ChIP-seq
	x Flow cytometry
x	MRI-based neuroimaging

Eukaryotic cell lines

Policy information about cell lines and Sex and Gender in Research

Cell line source(s)	U-2 OS Flp-In-TREx cell line: from Dr. Stephen C. Blacklow (Harvard Medical School), reported in Mol Cell Biol. 2006 Jun;26 (12):4642-51 (doi: 10.1128/MCB.01655-05)<br>Flp-In-TREx 293 cell line: Invitrogen (R78007)<br>HeLa Kyoto Flp-In cell line: from Dr. Amparo Andres-Pons (EMBL, Heidelberg)
Authentication	Cell lines were not further authenticated.
Mycoplasma contamination	All the cell lines generated/used in this study have been tested via PCR regularly and were negative.
Commonly misidentified lines (See ICLAC register)	No cell lines commonly misidentified were used in this study.

Plants

Seed stocks	N/A
Novel plant genotypes	N/A
Authentication	N/A

Flow Cytometry

Plots

Confirm that:

- [x] The axis labels state the marker and fluorochrome used (e.g. CD4-FITC).
- [x] The axis scales are clearly visible. Include numbers along axes only for bottom left plot of group (a 'group' is an analysis of identical markers).
- [x] All plots are contour plots with outliers or pseudocolor plots.
- [ ] A numerical value for number of cells or percentage (with statistics) is provided.

Methodology

Sample preparation	Sample preparation procedures for flow cytometry and FACS analysis are detailed in the corresponding Methods sections.
Instrument	BD Fortessa X-20 Cell Analyzer, BD FACSMelody Cell Sorter
Software	FlowJo (version 10.7.2)
Cell population abundance	For yeast surface display, the top performing cells (~1% of the total events) were sorted and cultured for the next round of FACS sorting. The specific sorted counts are listed in Supplementary Table 12-13.

Gating strategy	Yeast and mammlian cells were hierarchically gated by live cell (SSC-A/FSC-A), singlets (FSC-H/FSC-A) and self-labeling protein labeling signal (label channel/expression channel). Detailed gating strategies are described in the corresponding Methods sections and in Supplementary Figure 10-11.

- [x] Tick this box to confirm that a figure exemplifying the gating strategy is provided in the Supplementary Information.
